# In *Arabidopsis thaliana* mitochondria 5′ end polymorphisms of *nad4L-atp4* and *nad3-rps12* transcripts are linked to RNA PROCESSING FACTORs 1 and 8

**DOI:** 10.1007/s11103-021-01153-9

**Published:** 2021-04-28

**Authors:** Sarah Schleicher, Stefan Binder

**Affiliations:** Institut Molekulare Botanik, Universität Ulm, Albert-Einstein-Allee 11, 89069 Ulm, Germany

**Keywords:** *Arabidopsis thaliana*, Mitochondria, RNA processing, 5′ End polymorphisms, Pentatricopeptide repeat proteins

## Abstract

**Key message:**

RNA PROCESSING FACTORs 1 AND 8 (RPF1 and RPF8), both restorer of fertility like pentatricopeptide repeat proteins, are required for processing of dicistronic *nad4L-atp4 *and *nad3-rps12 *transcripts in Arabidopsis mitochondria.

**Abstract:**

In mitochondria of *Arabidopsis thaliana* (Arabidopsis), the 5′ termini of many RNAs are generated on the post-transcriptional level. This process is still poorly understood in terms of both the underlying mechanism as well as proteins required. Our studies now link the generation of polymorphic 5′ extremities of the dicistronic *nad3-rps12* and *nad4L-atp4* transcripts to the function of the P-type pentatricopeptide repeat proteins RNA PROCESSING FACTORs 8 (RPF8) and 1 (RPF1). RPF8 is required to generate the *nad3-rps12* -141 5′ end in ecotype Van-0 whereas the *RPF8* allele in Col has no function in the generation of any 5′ terminus of this transcript. This observation strongly suggests the involvement of an additional factor in the generation of the -229 5′ end of *nad3-rps12* transcripts in Col. RPF1, previously found to be necessary for the generation of the -228 5′ end of the major 1538 nucleotide-long *nad4* mRNAs, is also important for the formation of *nad4L-atp4* transcripts with a 5′ end at position -318 in Col. Many Arabidopsis ecotypes contain inactive *RPF1* alleles resulting in the accumulation of various low abundant *nad4L-atp4* RNAs which might represent precursor and/or degradation products. Some of these ecotypes accumulate major, but slightly smaller RNA species. The introduction of RPF1 into these lines not only establishes the formation of the major *nad4L-atp4* dicistronic mRNA with the -318 5′ terminus, the presence of this gene also suppresses the accumulation of most alternative *nad4L-atp4* RNAs. Beside RPF1, several other factors contribute to *nad4L-atp4* transcript formation.

**Supplementary Information:**

The online version of this article contains supplementary material available (10.1007/s11103-021-01153-9).

## Introduction

Higher plant mitochondria contain DNA encoding several components essential for their crucial function in the cellular context and thus for plant fitness and viability (Gualberto and Newton 2017; Hammani and Giegé 2014). For the expression of this limited but important genetic information, the organelles support a comprehensive and complex protein network. Plant mitochondrial DNA is transcribed by phage-type single subunit RNA polymerases that can be exclusively found in mitochondria or targeted to both mitochondria and chloroplasts (Hedtke et al. 1997, 2000; Liere et al. 2011). Transcription is initiated at multiple conserved and non-conserved promoters (Binder et al. 1995; Kühn et al. 2009, 2005; Tracy and Stern 1995), which together with extensive post-transcriptional processes (see below) contribute to the generation of often very complex transcript pattern seen in many different plant species (Choi et al. 2012; Forner et al. 2007; Hammani and Giegé 2014). Another remarkable feature of seed plant mitochondria is the outstanding role of pentatricopeptide repeat (PPR) proteins both in terms of function and number. *Arabidopsis thaliana* (Arabidopsis) encodes approximately 450 PPR proteins with functions in virtually all aspects dealing with RNA but with particularly prominent roles in post-transcriptional processes of gene expression (Barkan and Small 2014; Gutmann et al. 2020; Schmitz-Linneweber and Small 2008). These proteins are characterized by poorly conserved about 35 amino acid-long motifs (PPR), which differ slightly in size and structure (Small and Peeters 2000). On the basis of motif composition, PPR proteins are divided into two major subclasses. The PLS-class proteins are composed of canonical P, short S, long L motifs and of optional extra C-terminal distinctive domains called E, E + and DYW (Cheng et al. 2016; Lurin et al. 2004). Members of this subclass are predominantly involved in RNA editing contributing to the selection of cytidines to be edited to uridines in chloroplasts and mitochondria (Small et al. 2020; Takenaka et al. 2014). P-class PPR proteins are characterized by a sequence of canonical P motifs, sometimes interspersed by other PPR motifs, containing more or less non-conserved amino acid sequences both at the N- and C-termini, respectively. This protein class fulfills important roles in intron splicing and post-transcriptional 5′ and 3′ end processing of transcripts from chloroplasts and mitochondria (Barkan and Small 2014; Binder et al. 2013; Rovira and Smith 2019; Wang et al. 2017, 2018). The function of PPR proteins in all these processes is based on sequence-specific binding to RNA, which is governed by the combinatorial amino acid code for the interaction of the PPR motifs with specific nucleotides. It has been suggested that certain amino acids at positions 5 and 35 within each repeat are in direct contact with distinctive nucleotides, however, the code is kind of degenerative since some amino acid combination might fit with different nucleotide identities (Barkan et al. 2012; Barkan and Small 2014; Cheng et al. 2016; Yagi et al. 2013; Yan et al. 2019). In addition, there are still many amino acid combinations to which binding preferences have not been assigned yet. Whether P-class proteins have additional functions is unclear at present, but they apparently do not have any enzymatic activity. In chloroplasts for instance, binding of PPR proteins block progression of 5′ and/or 3′ exonucleases thereby defining the extremities of the transcripts (Pfalz et al. 2009). In mitochondria, also two PPR proteins have been described, which suggest a homologous mechanism for 3′ end formation in this organelle (Haïli et al. 2013; Wang et al. 2017). In contrast, a different scenario emerges for the post-transcriptional generation of 5′ transcript termini. So far, no mitochondrial 5′ exonuclease has been described, whereas hints towards an endonucleolytic generation of such ends have been found in at least some instances. In addition, PPR proteins that participate in post-transcriptional generation of 5′ ends, called RNA PROCESSING FACTORs, are predicted to bind upstream of the generated termini (Binder et al. 2016). Most of the so far identified factors belong to a group of PPR proteins, which are highly similar to restorer of fertility proteins identified in other plant species, where they restore cytoplasmic male sterility (Dahan and Mireau 2013; Fujii et al. 2011). These proteins are characterized by high sequence variability and and many of them contain a conserved 25 amino acid-long C-terminus. Whether these proteins have additional molecular functions apart from RNA binding is unclear at present, as is the involvement of other proteins in 5′ transcript processing.

Towards a comprehensive characterization of proteins required for the post-transcriptional generation of 5′ ends of RNAs encoded in seed plant mitochondria, we now took advantage of natural genetic variation resulting in 5′ end polymorphisms observed for the dicistronic transcripts of *nad3-rps12* and *nad4L-atp4*. By applying linkage analysis, complementation tests and CRISPR-Cas9-induced mutant analysis, we identified RPF8 and RPF1 to be involved in the generation of distinct 5′ ends. Moreover, we found that alternative 5′ termini of these transcripts are generated independently from the here described factors, strongly suggesting that multiple factors can be engaged in the generation of RNAs with distinct 5′ ends for a given mitochondrial gene or cistron.

## Results

### Detection of a *nad4L-atp4* transcript polymorphism in Arabidopsis ecotypes

**Fig. 1:**
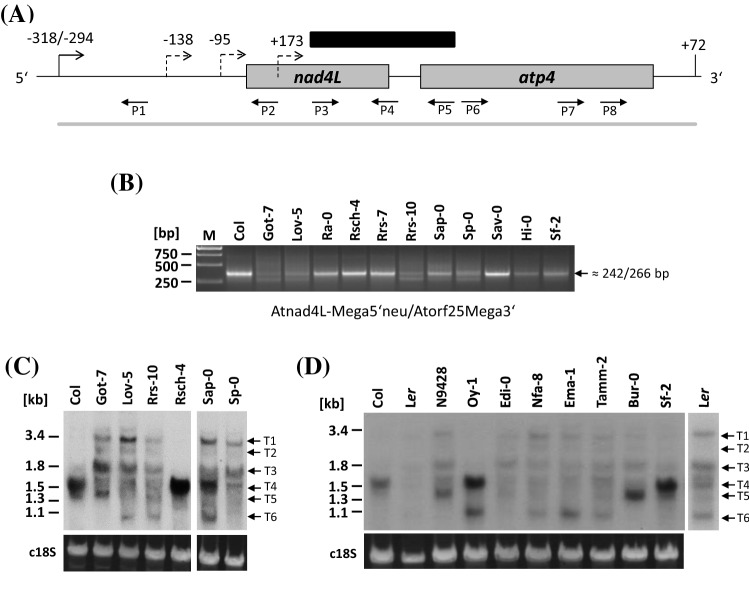
Investigation of *nad4L-atp4* transcripts in different Arabidopsis ecotypes. **a** Schema of the Arabidopsis *nad4L*-*atp4* genes (grey boxes). Black bent arrows and vertical lines mark previously mapped 5′ ends and 3′ ends, respectively, in Arabidopsis ecotype Col (Forner et al. 2007). New ends identified in Bur-0 (-138 and -95) and in Oy-1 (+ 173) are indicated as hatched bent arrows. Positions are given with respect to the translation start codon of *nad4L* (NATG, N = -1 for the 5′ ends) and relative to the translation stop codon of *atp4* (TAAN, N =  + 1, 3′ ends). A transcript found in Col is represented by a grey line, the probe used for Northern hybridizations is indicated as a black bar. Schema not drawn to scale. Oligonucleotides are indicated as horizontal arrows and designated: P1 Atnad4L-Mega.5‘neu, P2 Atnad4L-2, P3 Atnad4L-4, P4 Atnad4L-8, P5 Atorf25-2, P6 Atorf25-7, P7 Atorf25-4, P8 Atorf25-Mega.3’. Oligonucleotide sequences are listed in Supplemental Table S1. **b** CR-RT-PCR analysis. Primers used for PCR are given below the image. Products sizes expected in Col are indicated in the right margin. The sizes of DNA marker fragments are given in base pairs [bp]. **c** and **d** Northern blot hybridization of total RNAs from different Arabidopsis ecotypes and CSS line N9428 with a probe covering sequences from *nad4L-atp4* genes (indicated in Fig. 1a). At least six transcripts can be discriminated (T1 to T6), which are marked on the right-hand sides. Loading control (shown in the lower panels) was done by hybridization of oligonucleotide FAM-P18SRNAlong to cytosolic 18S rRNA (c18S). Sizes of various rRNA species are given in kilobases [kb] on left-hand side

In our efforts to identify and characterize proteins required for transcript maturation in plant mitochondria, we used circularized RNA (CR)-RT-PCRs to screen various Arabidopsis ecotypes for 5′ and 3′ end polymorphisms of *nad4L-atp4* mRNAs (Fig. 1b). In Col, amplification with primers Atnad4L-Mega5‘neu and Atorf25Mega3’ generated a product of about 270 bp which is consistent with the previously mapped 5′ and 3′ ends at position -318 and + 72 (Fig. 1a) (Forner et al. 2007). The same product is also amplified in other ecotypes strongly suggesting them to generate *nad4L-atp4* mRNA termini identical to Col. In contrast, in Got-7, Lov-5, Rrs-10, and Sp-0, a different pattern with two weak products was observed, while Sap-0, Hi-0 and Sf-2 showed ambiguous results (Fig. 1b). The size of the larger product was identical to the one in Col, while the other is at least 50 bp smaller. To confirm that the distinct PCR product pattern originated from different *nad4L-atp4* transcripts, a Northern blot analysis was performed with a probe amplified with primer pair Atnad4L-4/Atorf25-2 covering parts of both reading frames as well as the intergenic region and total RNA from Col, Got-7, Lov-5, Rrs-10, Rsch-4, Sap-0, and Sp-0. In Col and Rsch-4, transcripts of about 1,500 nucleotides (nt) were detected, matching the size of the 1,538 nt calculated from the above mentioned 5′ and 3′ termini in Col (Fig. 1c, transcript T4). In contrast, this RNA species was present only in minor amounts in Got-7, Lov-5, Rrs-10, and Sp-0 and at intermediate levels in Sap-0, instead additional larger and also smaller RNAs were found more abundant than in Col and Rsch-4 (Fig. 1c, transcripts T1 to T3, T5 and T6). Thus, the result of the Northern blot hybridization confirmed the presence of a *nad4L-atp4* transcript polymorphism in Arabidopsis ecotypes.

### The protein required for formation of the approximately 1,500 nt-long *nad4L-atp4* transcript is encoded on the upper part of chromosome 1

The Northern blot analysis of *nad4L-atp4* transcripts from the various ecotypes did not only reveal pattern different from Col, it also indicated minor differences among those ecotypes with “aberrant” *nad4L-atp4* RNA pattern*.* While a transcript species of about 1,400 nt is present in Got-7 (Fig. 1b, T5), Lov-5 and Rrs-10 contain comparatively small amounts of this RNA species but accumulate an additional small RNA of approximately 1,050 nt (Fig. 1c, T6). On the other hand, in Sap-0 intermediate amounts of the approximately 1,500 nt mRNA were detected in addition to an aberrant pattern of smaller or larger RNAs. This result revealed a remarkable variability of *nad4L-atp4* transcripts among Arabidopsis ecotypes. In addition, we realized that some of the ecotypes with “aberrant” *nad4L-atp4* transcripts have previously been found to be defective in generating the -228 5′ end of *nad4* transcripts (Hölzle et al. 2011). This observation suggested a link between these mitochondrial mRNA polymorphisms with both being connected to the same gene or to different genes located in close vicinity to each other in the nuclear genome. To test this assumption, we performed another Northern blot analysis including the ecotype L*er* and the single recombinant line N9428, which contains the Col nuclear genome except for the upper part of chromosome 1 (0–24 CM), which originates from L*er* (Koumproglou et al. 2002). This region encodes several genes for restorer of fertility-like (RFL) pentatricopeptide repeat (PPR) proteins. Both lines are impaired in *nad4* -228 5′ end processing (Hölzle et al. 2011). In addition, a number of other ecotypes were investigated representing different genotype clusters formed on the basis of 341 single nucleotide polymorphisms (Simon et al. 2012). The hybridization was done with the same probe as used in the above described Northern analysis and total RNA from the different strains. In L*er*, Edi-0, Nfa-8, Ema-1 and Tamm-2, the typical 1,538 nt-long dicistronic *nad4L-atp4* transcript was present in very low amounts or even absent (Fig. 1d, T4). Instead, other RNA species were detected, among them the above mentioned approximately 1,050 nt-long transcript that is present in different amounts (Fig. 1d, T6). This RNA species was also detected in Oy-1 in addition to the “normal” 1,538 nt-long dicistronic *nad4L-atp4* transcript as it is found in Col. In N9428 and Bur-0, also comparatively low amounts of the 1,538 nt-long RNA were seen. Instead, an approximately 1,400 nt RNA was found to be most prominent (Fig. 1d, T5). These results corroborated the strong variability of *nad4L-atp4* RNAs among Arabidopsis ecotypes. In addition, the absence of the 1,538 nt-long dicistronic *nad4L-atp4* transcript from N9428 suggested that the gene required to generate this transcript is encoded in the upper part of chromosome 1, most likely in a previously described gene cluster encoding RFL PPR proteins including RPF1 (Desloire et al. 2003; Fujii et al. 2011; Hölzle et al. 2011).

### RPF1 (At1g12700) is required for the formation of *nad4L-atp4* transcripts with -318 5′ termini

**Fig. 2:**
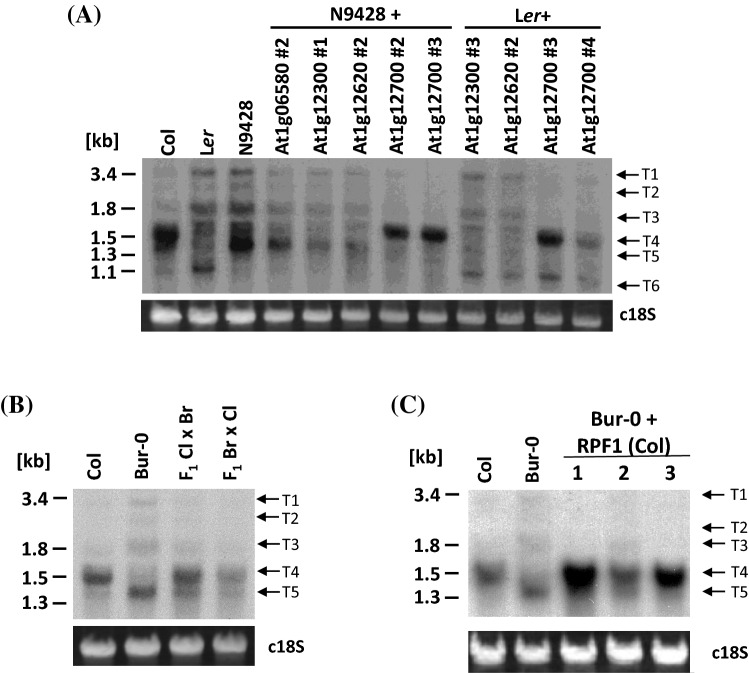
In vivo complementation assays. **a** Line N9428 and ecotype L*er* were transformed with different genes encoding restorer of fertility-like PPR proteins and total RNAs from individual plants were analyzed by Northern hybridization (AGI numbers given above the image). **b** *nad4L-atp4* transcript pattern in F_1_ hybrid plants obtained from reciprocal crossings of Col (Cl) and Bur-0 (Br). **c** Introduction of the *RPF1* allele into Bur-0. Three different plants (1–3) derived from three independent transformation events were investigated. Other descriptions see legend of Fig. 1

To identify this gene, we used previously established L*er* and N9428 lines, respectively, containing the Col alleles of At1g12300, At1g12620 and At1g12700 (*RPF1*) (Hölzle et al. 2011). In addition, we cloned the RFL PPR gene At1g06580 into pMDC123 (Curtis and Grossniklaus 2003) and introduced it into N9428. Then a Northern blot analysis of total RNA extracted from L*er* and N9428 plants containing the different Col alleles was performed. In those plants containing the genes At1g12300, At1g12620 and At1g06580, the 1,538 nt dicistronic *nad4L-atp4* transcript was present in very low amounts or absent and the detected pattern of RNAs was found identical with the original untransformed L*er* and N9428 lines (Fig. 2a). In contrast, in those plants containing the Col allele of At1g12700 (*RPF1*), the 1,538 nt *nad4L-atp4* transcript accumulated to amounts comparable to the Col wild-type control (Fig. 2a, T4). This result unambiguously demonstrated that *RPF1* is required for the accumulation of this transcript species. Interestingly, the amount of the 1,400 nt transcript detected at relatively high amounts in N9428 plants is markedly reduced when these plants contain an *RPF1* allele from Col (Fig. 2a, T5). Based on the combinatorial amino acid code for the interaction of the PPR motifs with specific nucleotides (one repeat one nucleotide code) we predicted (a) binding site(s) for RPF1 located between position -482 and -496 (in relation to the translation start codon of *nad4L*, NATG, N = -1) approximately 160 nt upstream of the -318 5′ end of the *nad4L-atp4* transcript (Supplemental Fig. S1a).

### The absence of the 1,538 nt dicistronic *nad4L-atp4* transcript correlates with the presence of nonsense stop codons in *RPF1* alleles

We have previously found that the defective processing at the *nad4* -228 5′ end is caused by a nonsense stop codon present in L*er* and other ecotypes (Hölzle et al. 2011). Thus we checked the sequence of the At1g12700 alleles from the here investigated ecotypes on genome browser web page of the Arabidopsis 1001 genome project (http://signal.salk.edu/atg1001/3.0/gebrowser.php). As expected, defective At1g12700 alleles with a premature stop at position G4,325,409A correlated with the absence of the 1,538 nt dicistronic *nad4L-atp4* transcript (Supplemental Fig. S2). Moreover, the alleles from Nfa-8 and Ema-1 contained several non-sense stop codons, one located further upstream locating the premature stop in the fourth PPR motif according to the redefined structures of the PPR proteins in Arabidopsis (Cheng et al. 2016). In Sap-0, the strongly reduced amount of the 1,500 nt *nad4L-atp4* RNA accompanied by the enhanced accumulation of larger and smaller transcript species does not correlate with any single nucleotide polymorphism, since no sequence deviations with respect to Col are given for this ecotype. In addition, the sequences of Rrs-7 and Rsch-4 exhibit several amino acid differences in comparison to Col (Supplemental Fig. S2). In both ecotypes, the amino acid identity at position 35 of PPR motif 1 is Gly (instead of Ser in Col), however, according to the combinatorial code, no binding of resulting amino acid combinations is predicted at this position in Col, Rrs-7 and Rsch-4, which is consistent with identical *nad4L-atp4* transcript phenotypes in these ecotypes (Fig. 1b and c).

### 5′ Ends of the shorter *nad4L-atp4* transcripts in Bur-0 and Oy-1

In Bur-0, an approximately 1,400 nt-long mRNA is the predominant *nad4L-atp4* transcript, which was also seen in N9428, but not in L*er*, whereas in Oy-1 an about 1,050 nt-long RNA was detected in addition the Col-typical 1,538 nt dicistronic *nad4L-atp4* transcript. To determine the extremities of the mRNAs in Bur-0 and Oy-1, we performed a CR-RT-PCR with primers Atnad4L-5 and Atorf25-7. This generated cDNA products of about 600 bp in all ecotypes. A sequence analysis of these products from Bur-0 and Oy-1 identified 5′ ends at a position -318 as observed previously in Col (Supplemental Figs. S3a and S4a) (Forner et al. 2007). Another CR-RT-PCR approach with oligonucleotide Atnad4L-2 annealing to sequences at the 5′ end of the *nad4L* reading frame and Atorf25-7 amplified products of about 900 bp in Col and Oy-1. These products also correspond to 5′ termini at -318. In Bur-0, products of about 650 and 700 bp, respectively, were generated, whose sequence analysis mapped new 5′ ends to positions -138 and -95, respectively (Supplemental Fig. S3b). Applying another primer combination (Atnad4L-8/Atorf25-4) for the detection of extremities of smaller RNAs generated a cDNA product of approximately 350 bp in Oy-1, which after sequence analysis was assigned to a 5′ terminus at position + 173 within the *nad4L* gene (Supplemental Fig. S4b). In all products analyzed, the 3′ ends were found at position + 72, as exemplarily shown for the 650 bp product amplified with primer pair Atnad4L-2/Atorf25-7 in Bur-0 (Supplemental Fig. S3c). This result was consistent with 3′ end determined in a previous study in Col (Forner et al. 2007). From these data, sizes of *nad4L-atp4* transcripts can be calculated with 1,538 and 1,046 nt in Oy-1, respectively, and 1,358/1,315 nt in Bur-0. These sizes fit well with transcripts T4 and T6 in Oy-1 and T5 in Bur-0 (Fig. 1d). In Oy-1, the -318 5′ end was also detected which originated from transcript species T4 (Fig. 1d). In summary, the results from the Northern hybridization and the CR-RT-PCR analyses demonstrated the extreme *nad4L-atp4* transcript diversity in Arabidopsis mitochondria.

### In Bur-0 and Oy-1, the generation of the shorter *nad4L-atp4* transcripts is independent from RPF1

Both, the ecotype L*er* and the single recombinant line N9428 do not accumulate the Col-typical 1,538 nt *nad4L-atp4* transcript to substantial amounts, which is linked to the presence of an inactive *RPF1* allele with a nonsense stop codon. But in contrast to L*er*, N9428 contains an approximately 1,400 nt-long *nad4L-atp4* mRNA, which is identical to the one observed in Bur-0 (Fig. 1d). The generation of this transcript species seems to be independent from *RPF1*, as suggested by the differential accumulation in L*er* and N9428. In addition, the introduction of the *RPF1* allele from Col into N9428 did not only establish the generation of Col-typical 1,538 nt *nad4L-atp4* transcript, it also lowered the accumulation of the 1,400 nt-long *nad4L-atp4* mRNA species (Fig. 2a). To check whether this phenomenon holds also true for the 1,400 nt-long transcript in Bur-0, we performed reciprocal crossings between Col and Bur-0. A Northern blot analysis revealed identical pattern of the reciprocal F_1_ hybrids. In both plants, the larger 1,538 nt transcript accumulates to slightly higher amounts than the smaller one, when compared to the parental ecotypes, suggesting the generation of these transcripts influence each other (Fig. 2b). In addition, we also introduced the Col allele of *RPF1* into Bur-0. A Northern analysis of three transgenic plants showed again that the generation of the Col-typical 1,538 nt *nad4L-atp4* transcript is linked to the Col allele of *RPF1* and that the presence of this allele markedly reduced the accumulation of the shorter 1,400 nt-long transcript (Fig. 2c).

In contrast to the 1,400 nt-long *nad4L-atp4* mRNA species, the generation of the roughly 1,050 nt transcript in Oy-1, which is also present in L*er* and other ecotypes, is completely independent from the generation of the other major mRNA. This is indicated by the introduction of the *RPF1* allele from Col into L*er*, which leads to the generation of the Col-typical 1,538 nt *nad4L-atp4* transcript but which does not interfere with the generation of the 1,050 nt RNA species. This observation is confirmed by the introduction of the *RPF1* allele from Oy-1 into L*er* and N9428. In both lines, the presence of this allele established the generation of the 1,538 nt *nad4L-atp4* transcript as observed in Oy-1 and Col. However, it neither interfered with the generation of the 1,050 nt RNA accumulation in L*er* nor did it induce the formation of this RNA species in N9428 (Supplemental Fig. S5).

Taken together, our studies demonstrate that multiple proteins participate in processing of *nad4L-atp4* mRNAs generating transcript species with different 5′ termini. Interestingly, the RPF1-dependent formation of the larger 1,538 nt transcript with the -318 5′ end is dominant over the generation of the smaller 1,400 nt-long *nad4L-atp4* mRNA species with the -139/-95 5′ termini, whereas the generation of the small 1,050 nt RNA species is independent from RPF1.

### Assigning the *RPF8* locus to the upper arm of chromosome 1

**Fig. 3:**
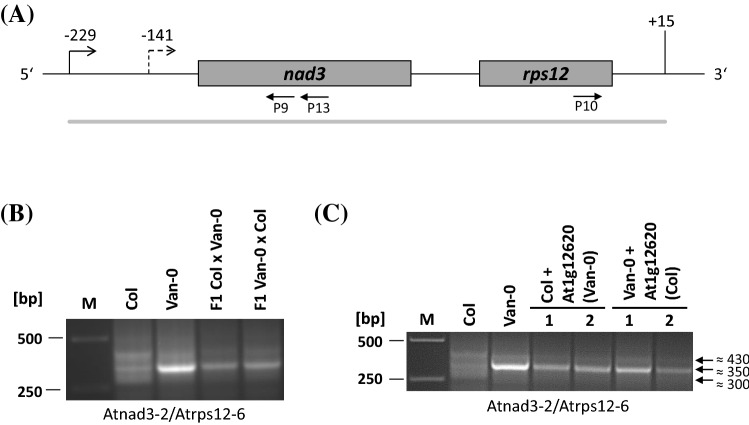
Identification of the *RPF8* gene. **a** Schema of the Arabidopsis *nad3*-*rps12* genes. A 5′ end identified in Van-0 (-141) is indicated as hatched bent arrow, a previously mapped 5′ terminus at -229 in Col is shown as black bent arrow. Positions are given with respect to the translation start codon of *nad3* (NATG, N = -1 for the 5′ ends) and relative to the translation stop codon of *rps12* (TGAN, N =  + 1, 3′ ends). Oligonucleotides indicated are: P9 Atnad3-2, P10 Atrps12-6 and P13 Atnad3-5. Oligonucleotide sequences are listed in Supplemental Table S1. Other descriptions see legend to Fig. 1. **b** CR-RT-PCR analysis of *nad3-rps12* mRNAs in Col, Van-0 and F_1_ plants obtained by reciprocal crossings between these ecotypes. **c** Analogous reciprocal investigations of Col and Van-0 plants transformed with alleles of At1g12620

In a previous study, we observed a polymorphism affecting the 5′ ends of the dicistronic transcripts of *nad3-rps12* genes (Stoll et al. 2013). In the accessions Col and Van-0, distinct 5′ termini at positions -229 (Col) and -140 to -142 (Van-0) were indicated by different CR-RT-PCR product pattern (Fig. 3a). To check whether the divergent 5′ ends in Col and Van-0 are linked to differences in nuclear or mitochondrial DNA, we performed reciprocal crosses between Col and Van-0. A CR-RT-PCR analysis of total RNA from the parental lines and the reciprocal Col/Van-0 F_1_ hybrids generated three products of 300, 350 and 430 bp in Col, whereas only a single product of 350 bp was seen in Van-0 and in the reciprocal F_1_ hybrids (Fig. 3b). These results demonstrated the biparental inheritance of the 5′ end polymorphism and the dominance Van-0 5′ end phenotype. Sequence analysis of the 350 bp products from Van-0 and Col and the 300 bp from Col identified 5′ termini at position -141 (350 bp product) in both ecotypes and -74 in Col (Supplemental Fig. S6a, b d and e). In addition, the + 15 3′ end was found in Van-0, which is consistent with formerly mapped 3′ terminus in Col (Supplemental Fig. S6c) (Forner et al. 2007). To map the genetic locus, which we called RNA PROCESSING FACTOR 8 (*RPF8*), linked to the *nad3-rps12* transcript polymorphism, we established a F_2_ mapping population (Col/Van-0) and performed a linkage analysis with F_2_ plants showing the recessive Col mRNA phenotype. Applying markers roughly covering the complete Arabidopsis nuclear genome, we localized the locus to a 1.58 Mbp genomic section on the upper arm of chromosome 1 (Jander et al. 2002)(Supplemental Fig. S7). This region contains several genes encoding restorer of fertility-like (RFL) PPR proteins (Fujii et al. 2011), some of which were found to be involved in other mitochondrial 5′ processing events as mentioned above (Fujii et al. 2011, 2016; Hölzle et al. 2011). Thus, an involvement of such a gene and its gene product in 5′ processing of *nad3-rps12* RNAs was considered in our further experiments.

### RPF8 is encoded by At1g12620

To unambiguously identify the gene involved in processes forming the 5′ ends of *nad3-rps12* transcripts, we cloned the Col and Van-0 allele of At1g12620 including 837 and 744 bp upstream and downstream regions into vector pMDC123 and introduced this gene into Col and Van-0 plants, respectively. When the Van-0 allele of At1g12620 was introduced into Col, a single product of 350 bp was generated by a CR-RT-PCR, consistent with the pattern of the Van-0 parental line and clearly different from Col (Fig. 3c). In the reciprocal experiment, the introduction of the Col allele of At1g12620 into Van-0 did not alter the CR-RT-PCR product pattern in the recipient ecotype, which is consistent with the recessive phenotype linked to the Col allele. Taken together, these results demonstrate that RFP8 is encoded by At1g12620 and the corresponding gene product is required for the generation of the − 141 (− 140 to − 142) 5′ end of the *nad3-rps12* transcripts in Van-0. Thus, another restorer of fertility-like PPR protein could be assigned to a plant mitochondrial 5′ processing event. A potential binding site for RPF8 was predicted to be located between nucleotide position − 173 and − 188 (with respect to the translation start codon of *nad3*, NATG, N = − 1), approximately 30 nt upstream of the − 141 5′ end (Supplemental Fig. S1b).

### The *RPF8* allele from Col does not have any function in *nad3-rps12* transcript maturation

**Fig. 4:**
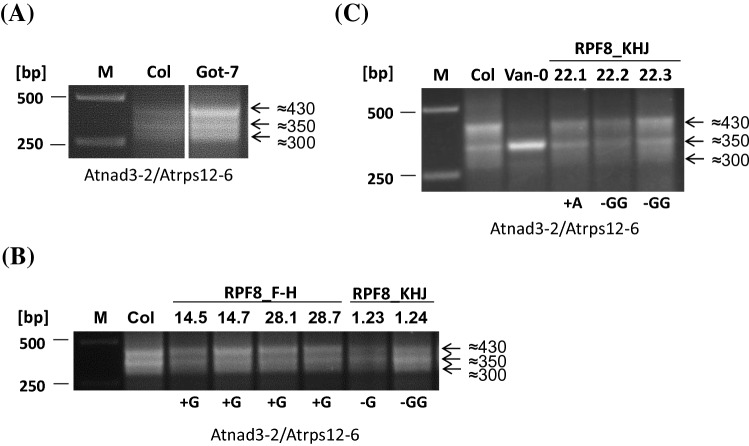
Allele-specific effects of *RPF8* knockouts on *nad3-rps12* mRNAs. CR-RT-PCR of ecotype Got-7 containing a natural *RPF8* knockout allele (**a**), of Col lines containing CRISPR-Cas9-induced frame shift knockout alleles (**b**) and of Van-0 lines with CRISPR-Cas9-induced frame shift mutations in *RPF8* (**c**). Inserted and deleted nucleotides are given below the individual lanes. Details of the alleles are given in Supplemental Fig. S9. Other descriptions see legend of Fig. 3

As demonstrated above, the Col allele of *RPF8* is dispensable for the efficient generation of *nad3-rps12* RNAs with -141 5′ ends, accordingly slightly larger dicistronic RNAs with -229 5′ termini accumulate in this ecotype (Stoll et al. 2013). Nevertheless, minor amounts of the shorter RNA species are detectable in Col, suggesting either a residual activity of the *RPF8* allele from Col or spontaneous generation of this transcript end as observed for other 5′ processing events (Binder et al. 2013) (Supplemental Fig. S6d). The *RPF8* genes from the analyzed ecotypes encode PPR proteins of 621 amino acids with 16 PPR motifs (Cheng et al. 2016; Yan et al. 2019). For comparison of these proteins, we sequenced the *RPF8* alleles from ecotypes with different *nad3-rps12* 5′ end phenotypes, respectively. These include Blh-1 and Got-7 with an *nad3-rps12* CR-RT-PCR product pattern identical to Col, Ra-0 identical to Van-0, and as well as Tsu-1 and La-1, whose *nad3-rps12* CR-RT-PCR product pattern deviate both from Col and Van-0 (Supplemental Fig. S8, bottom part) (Stoll et al. 2013). A comparison of the deduced amino acid sequences revealed several differences between the different alleles, some of them affecting the critical positions 5 and 35 within the PPR motifs (Supplemental Fig. S8). However, this analysis also identified a defective *rpf8* allele in Got-7, which contains a deletion at nucleotide position 667 (with respect to the ATG, A =  + 1) resulting in a truncated protein of 304 amino acids, which aligns to other RPF8 sequences up to amino acid position 225. Interestingly, this ecotype showed a *nad3-rps12* CR-RT-PCR pattern identical to Col (Fig. 4a), suggesting the *RPF8* allele from Col to be irrelevant for the generation of *nad3-rps12* transcripts with the -229 5′ termini. To further substantiate this conclusion and to further test the importance of the *RPF8* allele for the generation of *nad3-rps12* mRNAs in Van-0, we applied CRISPR-Cas9-based genome editing to generate *RPF8* knockout alleles in Col and Van-0. Several *RPF8* mutants in Col containing insertion or deletion of one or two nucleotides leading to premature stop codons in repeat 1 and repeat 9 (Supplemental Fig. S9), respectively, did not alter the CR-RT-PCR products pattern compared to Col wild type (Fig. 4b). In contrast to these results, the knockout of the Van-0 allele of *RPF8* in two different lines changed the *nad3-rps12* CR-RT-PCR product pattern from the Van-0 phenotype to the one seen in Col (Fig. 4c). These results confirmed the requirement of an intact *RPF8* allele for the efficient generation of *nad3-rps12* transcripts with − 141 5′ ends in Van-0, but also demonstrated that the RPF8 in Col is irrelevant for the formation of the dicistronic mRNAs with the − 229 5′ termini.

### Import of RPF8 into mitochondria

**Fig. 5:**
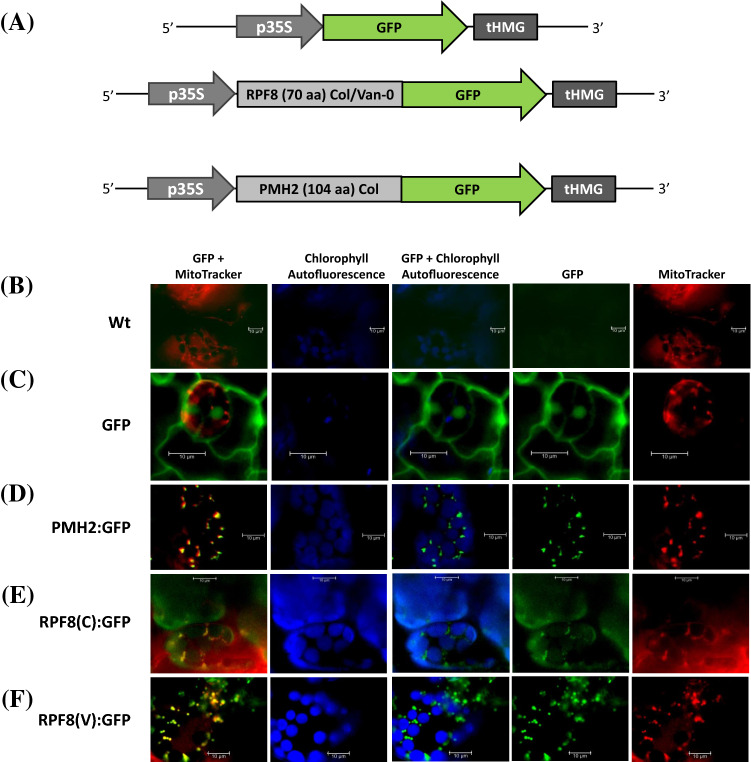
Subcellular localization of RPF8 protein from Col and Van-0. Epifluorescence microscopy of leaf samples obtained Arabidopsis Col wild type **a** Schematics of the constructs used for the GFP tagging experiments. **b** Col Wt, **c** GFP alone, **d** PMH2_1-104_:GFP, **e** RPF8(Col)_1–70_:GFP and **f** RPF8(Van-0)_1–70_:GFP fusion proteins. Fluorescence visible is indicated above the images. For each construct (indicated in the left), cells are investigated with different filter sets. The visualized fluorescences are given above the images. GFP and MitoTracker (first column) as well as GFP and chlorophyll autofluorescence (third column) are merged from images of the corresponding individual fluorescence images. Size standards (10 µm) are given in each image

To test whether the RPF8 protein is imported into mitochondria, we generated plant transformation constructs in vector pGGZ (Lampropoulos et al. 2013), in which the cDNA covering the N-terminal 70 amino acids of RPF8 from Col and Van-0, respectively, were N-terminally fused to the gene encoding the green fluorescent protein (RPF8(C):GFP and RPF8(V):GFP). An analogous construct containing the 104 N-terminal amino acids from the PUTATIVE MITOCHONDRIAL RNA HELICASE 2 (PMH2:GFP), a mitochondrial protein (Matthes et al. 2007) and a construct with a GFP gene without additional sequences (GFP) were used as positive and negative control, respectively (Fig. 5a). After transformation and selection of transgenic plants, leaf samples were inspected by epifluorescence microscopy. No GFP fluorescence was detected in wild-type plants, while GFP alone without additional amino acids attached was seen in the cytosol and the nucleus (Figs. 5b and c). As expected, the PMH2:GFP fusion protein is found in mitochondria as indicated by the identical pattern of GFP fluorescence (green) and the of MitoTracker staining (red) resulting in yellow colored organelles in the overlaid images (Fig. 5d). Analogous results were seen for the RPF8(C):GFP and RPF8(V):GFP fusion proteins (Fig. 5e and f), which strongly suggested that RPF8 localizes to mitochondria in both ecotypes. In addition, these experiments also demonstrated that a single amino acid difference in the RPF8 putative mitochondrial targeting sequences of the two ecotypes did not influence the subcellular targeting of the fusion proteins (Supplemental Fig. S8).

## Discussion

### Transcript diversity is a striking feature of Arabidopsis mitochondria

Arabidopsis and other plant species exhibit an enormous variability of mitochondrial transcript sizes and pattern for a given gene. So far, comprehensive studies in Arabidopsis and wheat revealed that this trait is almost exclusively linked to 5′ end heterogeneity whereas more or less all transcripts in a given species contain identical 3′ termini for a certain gene (Choi et al. 2012; Forner et al. 2007). The diversity of 5′ ends depends on a variance of the proteins involved in their formation as it is observed for the restorer of fertility-like PPR proteins involved in post transcriptional generation of 5′ extremities. These proteins are encoded by highly variable fast evolving genes that are present as different variants in different Arabidopsis ecotypes producing mitochondrial polymorphisms (Binder et al. 2016; Desloire et al. 2003; Forner et al. 2008; Fujii et al. 2011; Jonietz et al. 2010). This holds also true for the *nad4L-atp4* and *nad3-rps12* dicistronic transcripts and RPF1 and RPF8 involved in their 5′ end formation (Fig. 1).

### Different factors generate *nad4L-atp4* mRNAs

RPF1 was previously found to be required for 5′ end formation of *nad4* mRNAs. Now our studies demonstrate that this protein is also involved in processing of *nad4L-atp4* dicistronic transcripts. The absence of the 1,538 nt major *nad4L-atp4* mRNA in L*er* and in N9429 and the accumulation of this RNA species after the introduction of the *RPF1* allele from Col into these lines unambiguously demonstrates the crucial function of this protein for the formation of this dicistronic mRNA with the -318 5′ end (Fig. 2a). Interestingly, numerous Arabidopsis ecotypes contain a natural *RPF1* knockout allele (Supplemental Fig. S2)(Hölzle et al. 2011), which results in different *nad4L-atp4* mRNA phenotypes. In Bur-0, slightly smaller approximately 1400 nt-long transcripts with 5′ ends at positions -138/-95 are generated, which appear to be translatable mRNAs. These transcript species are also present in N9428 and in trace amount also in Col and their formation most likely requires another processing factor (Fig. 1). However, they were absent from many other ecotypes as for instance Tamm-2 or Ema-1, which most likely also lack this other processing factor and thus completely lack any prominent *nad4L-atp4* transcript species but instead exhibit very moderate amounts of many larger and smaller RNA ranging from 1.0 to 3.5 kb (Fig. 1). It seems that these transcripts are sufficient for translation of the corresponding proteins. The formation of smaller RNAs in the absence of the “normal” 1,538 nt-long mRNA indicates that the larger RNAs, which most likely represent precursor molecules, are susceptible to degradation. In contrast, when an intact *RPF1* allele is present, no other *nad4L-atp4* transcripts are detectable except for small amount of the 1,358 nt-long transcripts, which are also detectable in Col (Fig. 1c and d). This observation strongly suggests that RPF1 warrants not only an efficient formation of the -318 5′ end but at the same time supports the stability of the 1,538 nt major *nad4L-atp4* mRNA similar to what has been observed for RPF5 and RPF7 for 26SrRNA and *nad2* mRNA (Hauler et al. 2013; Stoll et al. 2014). In addition, the presence of the *RPF1* gene suppresses the accumulation of the shorter approximately 1,400 nt-long transcripts, which indicates that RPF1 is more efficient in 5′ processing than the factor(s) generating the -138/-95 termini and/or that the 1,538 nt major *nad4L-atp4* mRNA is not a substrate for the generation of these smaller dicistronic mRNAs (Fig. 2b and c). The relatively short 1,047 nt-long RNA, present in Oy-1 and other ecotypes exhibits a 5′ end within the *nad4L* reading frame and thus represents a monocistronic *atp4* RNA, but it might also be a degradation product. The generation of this transcript species is independent from RPF1 and from a potential factor required for the generation of the 1,400 nt mRNAs (Supplemental Fig. S5).

A categorization of the *nad4L-atp4* transcript phenotypes is very difficult as indicated by the results of Northern blot hybridization (Fig. 1c and d). However, when we inspected the *RPF1* alleles in the Arabidopsis 1001 genome project (The 1001 Genomes Consortium 2016)(http://signal.salk.edu/atg1001/index.php), about one fourth of the ecotypes contains a defective *RPF1* allele, strongly suggesting that all of these ecotypes show *nad4L-atp4* transcript pattern different from Col. This high percentage already indicates that ecotypes with non-functional *RPF1* alleles are found in all of the Arabidopsis ecotype clusters based on genotyping with nuclear single nucleotide polymorphisms (Simon et al. 2012).

Taken together our study demonstrates that different factors generate distinct 5′ ends at *nad4L-atp4* transcripts. RPF1 is clearly the key factor for the generation of a prominent and stable dicistronic 1,538 nt *nad4L-atp4* mRNA. About 160 nt upstream of the -318 5′ end of the *nad4L-atp4* mRNA, we could predict (a) binding site(s) for this protein located between position -482 and -496 (relative to the translation start codon of *nad4L*, NATG, N = -1, Supplemental Fig. S1a). Within the presumed sites, 12 or 13 interactions fit well with the suggested code for the interaction of PPR motifs with specific nucleotides, while only at the 5′ terminal and the 3′ third to last interactions deviate from the rules (Barkan et al. 2012; Cheng et al. 2016; Yagi et al. 2013; Yan et al. 2019). But compared to potential binding sites of other RPFs found about 30 to 80 nucleotides upstream of the affected 5′ end, the presumed site for binding of RPF1 to *nad4L-atp4* precursor RNAs is located relatively far upstream (Supplemental Fig. S1a). Although we have not checked the mitochondrial DNA sequences upstream of *nad4L*, an influence of mitochondrial DNA sequences can be excluded by the observed biparental inheritance of the mRNA phenotype seen in the reciprocal crosses between Col and Bur-0 as well as from the results seen in the complementation studies (Fig. 2).

### RPF8 is involved in 5′ processing of *nad3-rps12* mRNAs in Van-0 but not in Col

RPF8 is also a restorer of fertility-like PPR protein (Desloire et al. 2003; Fujii et al. 2011). This protein is involved in the generation of the -141 5′ ends of the *nad3-rps12* mRNA in Van-0, which is supported by the linkage analysis (Supplemental Fig. S7) and unambiguously demonstrated by the complementation assays (Fig. 3c) as well as the knockout of this gene in Van-0 (Fig. 4c). The -141 end is also detectable in Col, although the *RPF8* allele in this ecotype has no function in the generation of this end, as shown by the CR-RT-PCR analysis of the corresponding Col knockout mutant (Fig. 4b). The generation of minor amounts of distinct 5′ ends in the absence of cognate RPF has been observed also for other ends and might be the outcome of spontaneous processing by a potential ribonuclease at correct folded mRNA (Binder et al. 2013). The knockout of *RPF8* in Col also demonstrated that this gene is irrelevant for the formation of the -229 5′ terminus, which indicates that another factor is required for the generation of this end. Thus, several factors are engaged in 5′ processing of *nad3-rps12* dicistronic mRNAs.

Binding of RPF8 from Col and Van-0, respectively, was predicted to a site ranging from nucleotide positions -173 to -188 (relative to the translation start codon of *nad3*, NATG, N = -1), about 30 nt upstream of the -141 5′ end (Supplemental Fig. S1b). The mitochondrial DNA sequences up to 860 nucleotides of the *nad3* gene were found to be identical in Col and Van-0, except from a single insertion of a guanosine upstream of nucleotide position -641 in Van-0. However, mitochondrial DNA sequences are not responsible for the *nad3-rps12* mRNA polymorphism, which is shown by the biparental inheritance of mRNA phenotype in the reciprocal crosses (Fig. 3b). Within the predicted site, twelve (Van-0) and eleven (Col) amino acid/nucleotide contacts fit well with the suggested code for the interaction of PPR motifs with specific nucleotides, whereas the interactions at four (Van-0) and five (Col) contact sites, which are relatively uniformly distributed within the potential binding, deviate from the rules (Supplemental Fig. S1b). Thus, the prediction for RPF8 from Van-0 is slightly better than for the RPF8 protein from Col, however, it remains to be shown whether this minor differences are causative for the distinct functions of these proteins in the generation of the -141 5′ end of the *nad3-rps12* transcript.

Whether the *RPF8* allele from Col has another function is unclear at present, however, it is an intact gene with a complete reading frame coding for a protein with 96% identical amino acids when compared to the corresponding proteins in Van-0. Thus, another function appears likely. In addition, it remains also unclear whether the *RPF8* allele in Van-0 underwent a gain of function or whether the Col allele of *RPF8* encountered a loss of function. From the previously investigated 26 Arabidopsis ecotypes (Stoll et al. 2013), 19 were identical to Col, whereas only six showed slightly variable *nad3-rps12* RNA pattern different from Col. Similar to the distribution of the *nad4L-atp4* RNA phenotype, both, the group of ecotypes with Col *nad3-rps12* mRNA phenotype, as well as the ecotypes with aberrant transcript pattern can be found in different groups of ecotypes genotype-based clusters (Simon et al. 2012). The observed independent distribution of the phenotypes from the genotype cluster might be explained by the generally fast evolution of the restorer of fertility-like proteins.

The relatively small number of ecotypes with the *nad3-rps12* mRNA phenotype divergent from Col might argue for a gain of function in these ecotypes. However, small changes in the amino acid sequence may change the binding specificity of a PPR protein which makes the occurrence of loss of function more likely. Although there are some amino acid differences at position 5 and 35 between the alleles in Col and Van-0, the effect on predicted binding seems to be marginal suggesting amino acid differences at other positions to impact RPF8 function (Supplemental Fig. S1 and S8). The absence of a function of the *RPF8* allele from Col for processing of *nad3-rps12* transcripts in this ecotype might also be related to highly reduced expression of this gene, however, this assumption would also apply for most of the other ecotypes investigated, since these show *nad3-rps12* mRNA phenotypes identical to Col. Thus, this explanation seems to be rather unlikely. Since the GFP tagging experiments indicates that RPF8 from Col is imported into mitochondria (Fig. 5), mis-targeting of the protein can be excluded as a reason for missing relevance of this protein for *nad3-rps12* mRNA in this ecotype.

## Material and methods

### Plant cultivation

Arabidopsis ecotypes and single recombinant line N9428 were obtained from the Eurasian (Nottingham) Arabidopsis Stock Centre (NASC, http://arabidopsis.info/) or were a kind gift from Rhonda Meyer (IPK, Gatersleben, Germany). Arabidopsis plants were grown in a growth chamber on standard soil containing 20% Vermiculite grain size 2–3 mm (Isola-Mineralwolle-Werke GmbH, Germany) and 1.5 g/l Osmocote Exact Mini fertilizer (Scotts GmbH, Germany) or on Murashige and Skoog (MS) medium containing 0.5% (w/v) sucrose. Plant cultivation conditions were 16 h light (100–150 µmol/m^2^s)/8 h dark at 21° C. For selection of transformants on MS medium, seeds were germinated in the presence of 50 µg/ml hygromycin or 10 µg/ml Basta® (Sigma Aldrich, USA). For selection on soil, plants were sprayed three times with Basta®.

### Nucleic acid methods

Arabidopsis total RNA and DNA, respectively, was isolated from about two to four week-old plants using the Spectrum™ Plant Total RNA Kit (Sigma-Aldrich) or applying a previously established protocol (Edwards et al. 1991). CR-RT-PCR analyses and Northern blot hybridizations were performed as described before (Forner et al. 2007; Jonietz et al. 2010). The DNA probe applied in the Northern blot hybridization was generated by PCR with primer pair Atnad4L-4/Atorf25-2. The product was checked by agarose gel electrophoresis, excised and eluted from the gel using GenElute™ Gel Extraction kit (Sigma-Aldrich) and labeled by the incorporation of [α^32^P]dCTP using the DECAprime™ II DNA-Labeling Kit according to the recommendations given by the manufacturer (Thermo Fisher Scientific Inc.). Cytosolic 18S rRNA was detected with a 5′ FAM-labeled oligonucleotide (Sigma Aldrich) and results were recorded using a ChemiDoc MP Imaging System (Bio-Rad Laboratories, Inc). PCRs were done following standard PCR protocols with GoTaq DNA polymerase (Promega Corp.) or Phusion High-Fidelity DNA Polymerase (Thermo Fisher Scientific Inc.).

For the generation of *RPF8* knockout mutants, Arabidopsis plants were transformed with constructs containing the *Streptococcus pyogenes* CRISPR-Cas9 gene (adapted to plant codon usage) expressed under the control of the CaMV 35S promoter and a sgRNA expression cassette under the control of the Arabidopsis U6-26 promoter (pGGZ_SpCas9_sgRNA_Hyg). The latter contained either sgRNAs F (1663–1682, positions given with respect to ATG, A = 1), G (80–99) and H (930–949) or K (1129–1148), H and J (86–105), separated by Arabidopsis tRNA^Pro^. The cassettes containing three sgRNA-tRNA were assembled after amplification of four sgRNA-tRNA fragments with the following primer pairs: F–H: SP t start F/gRNA-F-At1g12620-R, gRNA-F-At1g12620-F/gRNA-G-At1g12620-R, gRNA-G-At1g12620-F/gRNA-H-At1g12620-R and gRNA-H-At1g12620-F/SP sg end R and KHJ: SP t start F/gRNA-K-At1g12620-R, gRNA-K-At1g12620-F/gRNA-H-At1g12620-R, gRNA-H-At1g12620-F/gRNA-J-At1g12620-R and gRNA-J-At1g12620-F/SP sg end R. The PCR products were cloned via a Greengate restriction/ligation reaction into the BpiI site in plasmid pGGZ_SpCas9_sgRNA_Hyg generating plasmids pGGZ_SpCas9_RPF8_F-H and pGGZ_SpCas9_RPF8_KHJ, respectively (Lampropoulos et al. 2013). Constructs were checked by DNA sequencing and subsequently transformed into Arabidopsis plants using Agrobacterium strain GV3101 containing pMP90 and pSOUP helper plasmids. After selection of transformed plants, mutations in the *RPF8* gene were identified and homozygocity in generation T_2_ or T_3_ confirmed by DNA sequencing. Sequencing was done commercially (Microsynth Seqlab, Germany). Digital analysis of nucleic acid sequences was done using SnapGene® Software (GSL Biotech LLC, USA). Oligonucleotide sequences are given in Supplemental Table S1. The approximate locations of *nad3-rps12* and *nad4L-atp4*-specific oligonucleotides are given in Figs. 1a and 3a.

### In vivo complementation assays

L*er* and N9428 plant lines containing complementation constructs for At1g12300, At1g12620 and At1g12700 were established previously (Hölzle et al. 2011). The nuclear gene encoding restorer of fertility like protein At1g06580 including 612 bp upstream and 919 bp downstream sequences was amplified from Col DNA with primer pairs At1g06580-Kompl.H/At1g06580-Kompl.R, digested with *Asc*I and *Pac*I and cloned into the corresponding sites in pMDC123 (Curtis and Grossniklaus 2003). At1g12620 including up to 837 bp upstream and 744 bp downstream sequences were amplified from Col and Van-0 DNA with primer pair At1g12620-Kompl.H/At1g12620-Kompl.R or At1g12620-Kompl.R2 and cloned into pMDC123 via *Asc*I and *Pac*I sites. The constructs were transformed as a mixture of at least three different clones to avoid effects of potential PCR errors. For complementation tests constructs were transformed into Arabidopsis plants using Agrobacterium strain GV2260 following the floral dip procedure (Clough and Bent 1998).

### Linkage analysis

F_1_ and F_2_ hybrid plants were generated according to standard procedures (Weigel and Glazebrook 2002). To map the locus containing the *RPF8* gene, an F_2_ mapping population was established from crossed Col x Van-0 F_1_ hybrids. Plants were phenotyped by CR-RT-PCR with oligonucleotide pair Atnad3-2/Atrps12-6 and 7 plants displaying the recessive Col phenotype were subsequently used for a linkage analysis using insertion/deletion markers (Supplemental Table S2). Results of the Col x Van-0 linkage analysis is summarized in Supplemental Fig. S7.

### Microscopy

For GFP-tagging experiments 210 nt DNA fragments corresponding to N-terminal *RPF8* sequences (70 amino acids) were amplified with primer pair At1g12620.GFP.fw/At1g12620.GFP.rev2 on Col and Van-0 total DNA, respectively, and cloned via the *BsaI* site into Greengate vector pGGB000 (pGGB_mtAt1g12620-Col, pGGB_mtAt1g12620-Van-0). The plant transformation plasmids were assembled in pGGZ003 in a Greengate reaction containing pGGA004 (CaMV 35S promoter), pGGB_mtAt1g12620-Col or pGGB_mtAt1g12620-Van-0, pGGC014 (GFP CDS), pGGD002 (Dummy C-tag), pGGE003 (HMG terminator) and pGGF008 (BastaR). Restriction/ligation reactions were done as described before (Lampropoulos et al. 2013). Likewise, a construct for the expression of PMH2:GFP fusion protein was assembled. To this end a 312 nt fragment DNA corresponding to 104 N-terminal amino acids of PMH2 was amplified with primer pair PMH2 fw/PMH2 rev1 and cloned into the *BsaI* site of pGGB000 (pGGB_PMH2).

Fluorescence microscopy was performed using a Leica DM5500 equipped with a Leica DFC3000 G digital camera with filter sets: Mitotracker: excitation 545/30 nm, beam splitter 570 nm, emission 610/75 nm; GFP: GFP excitation 480/40 nm, beam splitter 505 nm, emission 527/30 nm, and chlorophyll autofluorescence: excitation 470/40, beamsplitter 495, emission 525/50. Images were captured and processed with Leica LAS X software.

## Supplementary Information

Below is the link to the electronic supplementary material.

Electronic supplementary material 1 (PDF 130 kb)

Electronic supplementary material 2 (PDF 121 kb)

Electronic supplementary material 3 (PDF 1387 kb)
