# Supplementary material for: In Arabidopsis thaliana mitochondria 5′ end polymorphisms of nad4L-atp4 and nad3-rps12 transcripts are linked to RNA PROCESSING FACTORs 1 and 8
Source: Plant Mol Biol. 2021 Apr 28;106(4-5):335–48. doi: 10.1007/s11103-021-01153-9 (PMC8270843; doi:10.1007/s11103-021-01153-9)
Supplement: Supplementary file 1 — Electronic supplementary material 1 (PDF 130 kb) [file 11103_2021_1153_MOESM1_ESM.pdf]

Oligonucleotide	Sequence (5' to 3')
**CR-RT-PCRs**	
Atnad3-2	GGTAGGTAGAACTATTGGAAGC
Atnad3-5	AAGGATCGAAACCACATTCGTAGGCC
Atnad4L-2	AGGAGGATTCCCCGAATACC
Atnad4L-5	ACCTGCGAAAAAGAGCTTCC
Atnad4L-8	GGAATGGTTAACCTTGAATGC
Atnad4L-Mega5'neu	TTCGCTGCTGCGGCAAGG
Atorf25Mega3'	AGTAGAATTCATTCGAGAGAGCTTGGTGGTC
Atorf25-4	AATGCCACTTCTTCCCGTCG
Atorf25-7	GGCTTTATCATATTCAGTCG
Atrps12-6	AGGAGTAAAGGATTTGATGGGAATTCCGG
**Northern Probes**	
Atnad4L-4	AATCCGCTATTGGGTTAGCC
Atorf25-2	CATCTTTCTACCATCCATATTCG
FAM-P18SRNA long	[FAM]CGTCGCCAGCACAGAGGCCATGCGATCCGTCG
**Complementation Constructs**	
At1g06580-Kompl.H	AGTGCTGCGGCGCGCCCATATCATAGCTCTCGAGAACC
At1g06580-Kompl.R	ATATATTTAATTAAGTAGAAGCTAACGATAGACAC
At1g12300-Kompl.H	TGACTCCGAGGCTTAATTAACACACTGTGCTTGCCCAGCTAACCAC
At1g12300-Kompl.R	ATGCAGTCGAGCGGCGCGCCGGCACGATCTGTGACAAGGACTGTG
At1g12620-Kompl.H	ATGCAGTCGAGCGGCGCGCCGTTTTTGTCACGGGAGATGTATTATCAGG
At1g12620-Kompl.R	TGACTCCGAGGCTTAATTAATCCATGAAGATCTTGCTTGCAGAGTCC
At1g12620-Kompl.R2	TCGAACGTACTGTTAATTAAGTCCGAGTACAAGG
At1g12700-Kompl.H2	ATGCAGTCGAGCGGCGCGCCGAGCTCGTGTAAAGACCAGTTGAAGC
At1g12700-Kompl.R3	ATGGCATTAATTAAATTCAATAATGGTTTCAG
**Linkage analysis**	
CER453022.HA	CTATCCGAAATCCAAATTCCGGATG
CER453022.R	GATTCAAAAGTGGATCCCCATTGTACAG
Chr.1 3377847.H	GCAATAGTTTTGAAATTTGAGGG
Chr.1 3377847.R	ACAAATTCTAGCTGCCGTGC
Chr.1 3982507.H	CAAACATTGTTAATGAAAATGGTG
Chr.1 3982507.R	ACACAACATATTGAATACTCC
CER451941.H „neu“	GATTTCATCACTACATTCCAC

CER451941.R „neu“	TCTCTTCTATCCTTTACTCGTC
CER453516.H	TTAGAAATGAACAGGAGAATTGACTTATAGGACACAC
CER453516.R	CAATGATGTTGACTTTGAATACAAATTGGTAACC
Chr.1 21924745.H	ACAATTCCTTTGGCTGAGC
Chr.1 21924745.R	CAAAAGGATTATTGCTCGC
CER449403.H	TGATTCCAGCGGGTAGGCATGACC
CER449403.R	CGAAACATTCAAAAAGTGGTTATGGGAGAG
CER448906.H	GCACTTATGCTACCTTCTGC
CER448906.R	GATTTACATATGCCAATCCG
CER460534.H	CATGGTCAATGCAACGTTACAAAGTGC
CER460534.R	CGAAACGCCCAAAGCGTAATCTCC
CER456162.H	TTAGTAATGTGTAGGGTTCCC
CER456162.R	TGCTTCTCTATCTCCTATACTCTC
CER460934.H	CATGTGAAGAAGGAGACCCC
CER460934.R	ATTGAAGAAGAAGCCATGCC
CER458914.H	AAAGATTGAATGTGGCTGC
CER458914.R	ATAAGAGCACACATAAAAACCC
CER460528.H	CAATGATTTGGTGGGTCAAGAGTCAAGAC
CER460528.R	GCAAAAGTCATTACGGACAATACCAAACG
Chr.5 7044616.H „neu“	GGAATCTGAGAAAACATTCCC
Chr.5 7044616.R „neu“	CTGACAATCTGCCTGATACCG
CER449900.H	CCATCTACAATATTTAAAGAAGTCCC
CER449900.R	TTCTGAAGTTTTAATATCCTCGTCT
CER454081.H	GAATTATGTTCGTTGGAGC
CER454081.R	TTGTGATCTGCTTTTGCC
CER495904.H	CTAATTTGTTCGTGCAGAGT
CER495904.R	GCACACTTGTTTACATCCAC
**DNA Sequencing**	
At1g12620.A	TTTCCCTTTCCCATCACTGACAC
At1g12620.B	TCTCAGTTGATGCTTCCACGG
At1g12620.H	GCTTCGTTCTTCGTATCGATGTGTCTGG
At1g12620.H2	TAGCTTAAACACACCATCGC

At1g12620.H3	GATAAACGGATTATGTCTCGAG
At1g12620.H4	GATGCGGTCCTAATATCAGG
At1g12620-Kompl.H	ATGCAGTCGAGCGGCGCGCCGTTTTTGTCACGGGAGATGTATTATCAGG
At1g12620-Kompl.R	TGACTCCGAGGCTTAATTAATCCATGAAGATCTTGCTTGCAGAGTCC
At1g12620.R	TGCTTTCATGCCCGGAGAATGG
At1g12620.R2	TACCGGATCAGATAAAACCGG
At1g12620.R4	ACAGGTCTGCTTCAGACAGTGAGCC
At1g12620.R5	ACAGCATCAGAGACTTTACCG
At1g12620.R6	GGTTGGCCTTATCTAGCTGG
At1g12620.T1u2.H	GCTGATATTATTATCTACACCAC
At1g12620.T1u2.R	CACAAAACAATCTATTAACGCG
At1g12620.T3.H	GACAGGTACTCTAAGAACTGC
**sgRNA design**	
gRNA-G-At1g12620-F	ATATATGAAGACGTTGACACTAGAAAAGAGGGGCGTTCCGAGAATTG
gRNA-G-At1g12620-R	ATATATGAAGACGTGTCAGTGATGTTTTAGAGCTAGAAATAG
gRNA-H-At1g12620-F	ATATATGAAGACGTAATCACACCAGACGGTTTTAGAGCTAGAAATAG
gRNA-H-At1g12620-R	ATATATGAAGACGTGATTTTCCTTGGGGCGTTCCGAGAATTG
gRNA-J-At1g12620-F	ATATATGAAGACGTTCAGTGATGGGAAAGTTTTAGAGCTAGAAATAG
gRNA-J-At1g12620-R	ATATATGAAGACGTCTGACACTAGGGGGCGTTCCGAGAATTG
gRNA-K-At1g12620-F	ATATATGAAGACGTAGCAAAGGATGGTTTTAGAGCTAGAAATAG
gRNA-K-At1g12620-R	ATATATGAAGACGTTGCTAACCATCAGGGGGCGTTCCGAGAATTG
SP sg end R	ATATATGAAGACGCAAACAAAAAAAAAAGCACCGACTCG
SP t start F	ATATATGAAGACAAATTGGGGCGTTTGGTCTAGTG
**GFP tagging**	
At1g12620_GFP.fw	ATATATGGTCTCTAACAATGCGGGGATTGATTCAGAC
At1g12620.GFP.rev2	ATATATGGTCTCTAGCCAGGACGAGGACGTGACCTAGTCA
PMH2.fw	ATATATGGTCTCTAACAATGATCACTACAGTGCTACG
PMH2.rev1	ATATATGGTCTCTAGCCAAGCCCATCACCACCAACAC
