# Supplementary material for: In Arabidopsis thaliana mitochondria 5′ end polymorphisms of nad4L-atp4 and nad3-rps12 transcripts are linked to RNA PROCESSING FACTORs 1 and 8
Source: Plant Mol Biol. 2021 Apr 28;106(4-5):335–48. doi: 10.1007/s11103-021-01153-9 (PMC8270843; doi:10.1007/s11103-021-01153-9)
Supplement: Supplementary file 2 — Electronic supplementary material 2 (PDF 121 kb) [file 11103_2021_1153_MOESM2_ESM.pdf]

Supplemental Table 2: Markers

marker	chromosome/ position	oligonucleotides	product length Col	product length Van-0
CER453022	1/2.93 Mbp	CER453022.HA<br>CER453022.R	259 bp	≈240 bp
Chr.1 3377847	1/3.38 Mbp	Chr.1 3377847.H<br>Chr.1 3377847.R	281 bp	≈250 bp
Chr.1 3982507	1/3.98 Mbp	Chr.1 3982507.H<br>Chr.1 3982507.R	563 bp	≈350 bp
CER451941.H „neu“	1/4.51 Mbp	CER451941.H „neu“<br>CER451941.R „neu“	230 bp	≈180 bp
CER453516	1/5.30 Mbp	CER453516.H<br>CER453516.R	241 bp	≈210 bp
Chr.1 21924745	1/21.92 Mbp	Chr.1 21924745.H<br>Chr.1 21924745.R	317 bp	≈290 bp
CER449403	2/1.83 Mbp	CER449403.H<br>CER449403.R	229 bp	≈200 bp
CER448906	2/10.81 Mbp	CER448906.H<br>CER448906.R	300 bp	≈290 bp
CER460534	2/17.11 Mbp	CER460534.H<br>CER460534.R	543 bp	≈450 bp
CER456162	3/6.01 Mbp	CER456162.H<br>CER456162.R	349 bp	≈320 bp
CER460934	3/17.21 Mbp	CER460934.H<br>CER460934.R	387 bp	≈350 bp
CER458914	4/2.36 Mbp	CER458914.H<br>CER458914.R	221 bp	≈200 bp
CER460528	4/5.74 Mbp	CER460528.H<br>CER460528.R	278 bp	≈200 bp
Chr.5 7044616 „neu“	5/7.04 Mbp	Chr.5 7044616.H „neu“<br>Chr.5 7044616.R „neu“	392 bp	≈350 bp
CER449900	5/9.01 Mbp	CER449900.H<br>CER449900.H	220 bp	≈190 bp
CER454081	5/19.15 Mbp	CER454081.H<br>CER454081.R	417 bp	≈330 bp
CER495904	5/20.29 Mbp	CER495904.H<br>CER495904.R	530 bp	≈250 bp
