# Supplementary material for: In Arabidopsis thaliana mitochondria 5′ end polymorphisms of nad4L-atp4 and nad3-rps12 transcripts are linked to RNA PROCESSING FACTORs 1 and 8
Source: Plant Mol Biol. 2021 Apr 28;106(4-5):335–48. doi: 10.1007/s11103-021-01153-9 (PMC8270843; doi:10.1007/s11103-021-01153-9)
Supplement: Supplementary file 3 — Electronic supplementary material 3 (PDF 1387 kb) [file 11103_2021_1153_MOESM3_ESM.pdf]

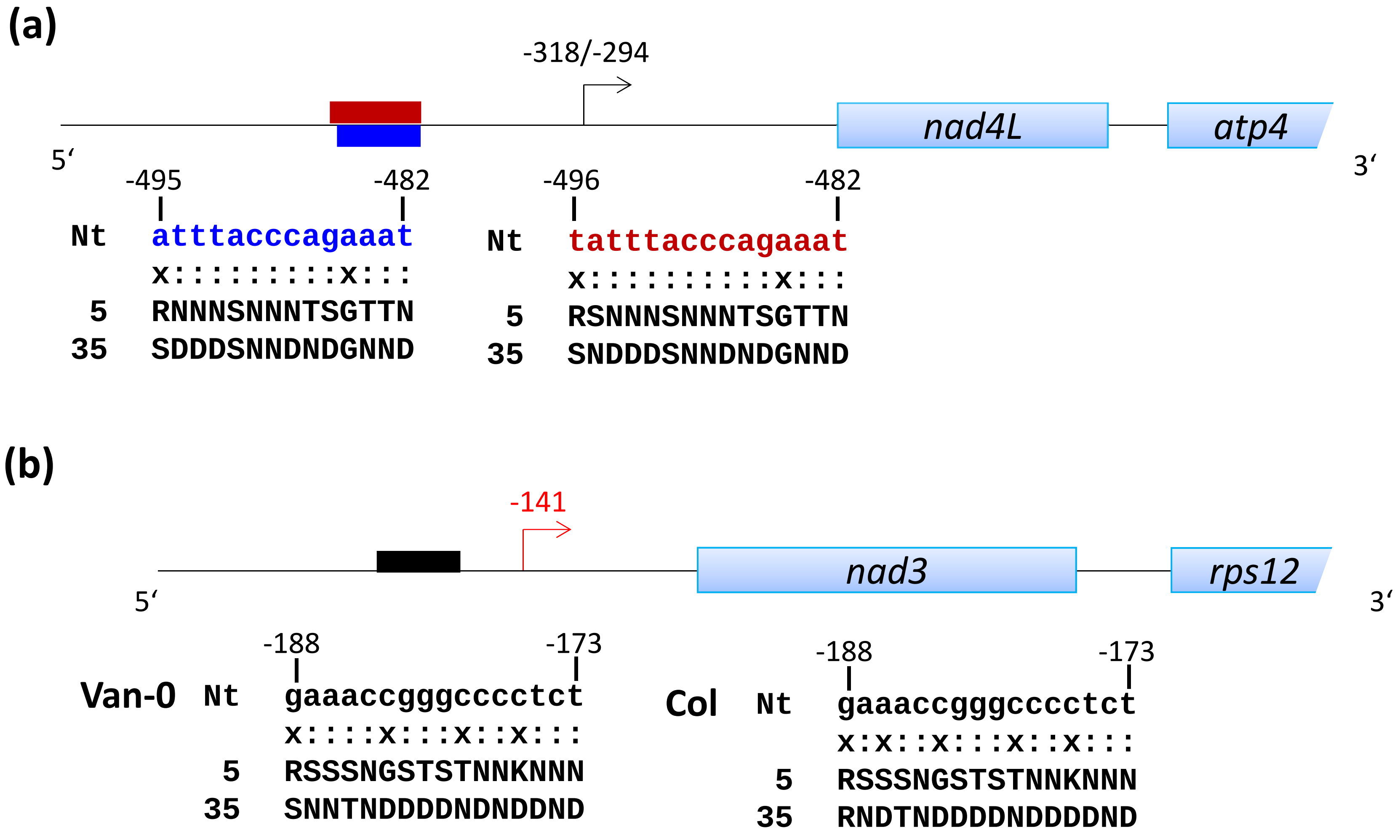

Supplemental Figure S1: Binding site predictions based on the combinatorial amino acid code for the interaction of the PPR motifs with specific nucleotides (one repeat one nucleotide code). **a** Two binding sites could be predicted for RPF1 upstream of the *nad4L-atp4* -318 5’ end. Theses sites differ by one nucleotide in length depending on Pi motif, which is included as a canonical P motif in the prediction given in red, whereas it is not included in the blue version. **b** Prediction for the binding of the RPF8 proteins from Van-0 and Col upstream of the -141 5’ terminus of the *nad3-rps12* transcript.

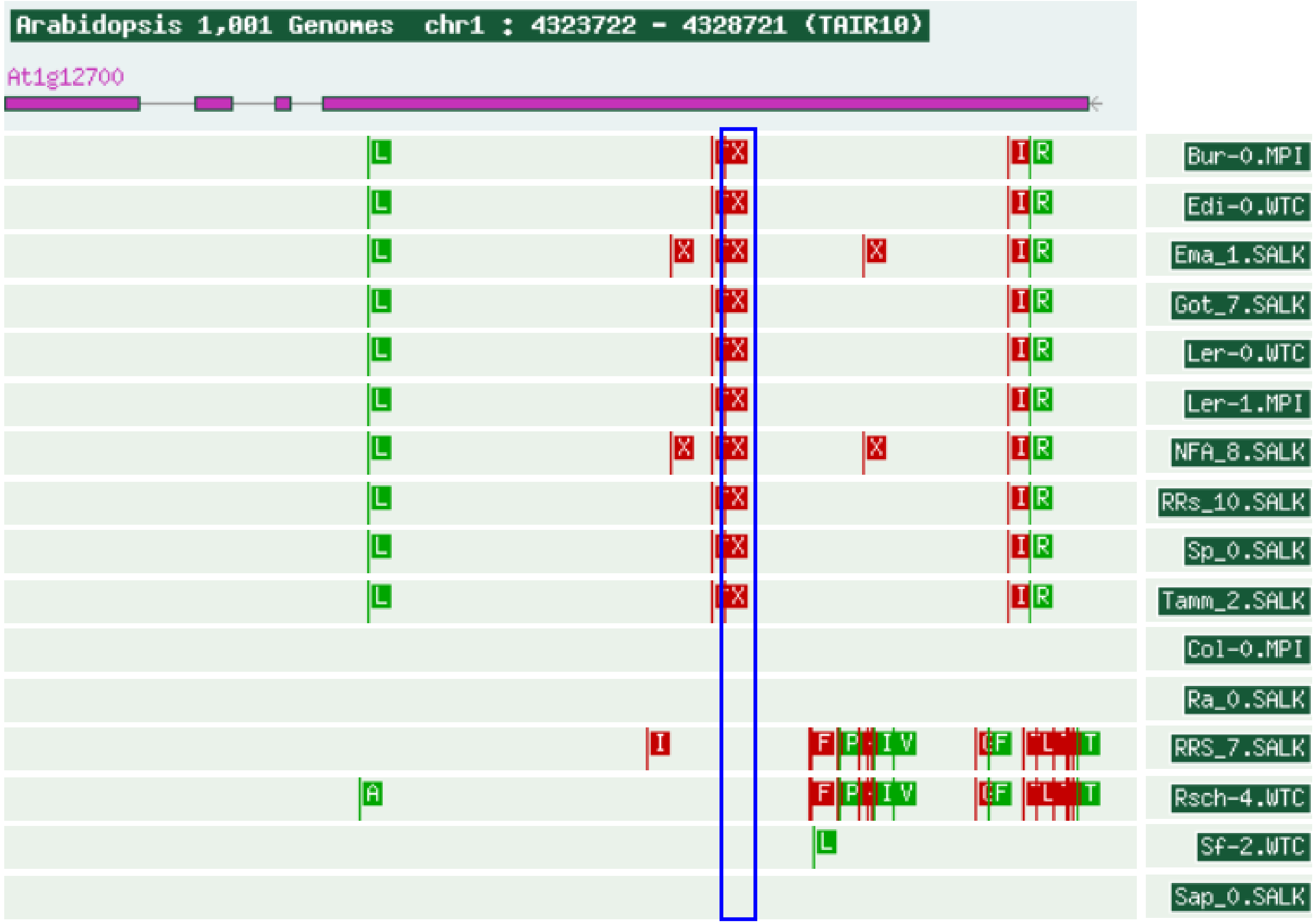

Supplemental Figure S2: Comparison of *RPF1* alleles from ecotypes with clear results concerning the presence of the 1,538 nt dicistronic *nad4L-atp4* transcript based on data from the 1001 genome project (http://signal.salk.edu/ atg1001/3.0/gebrowser.php). Ecotypes shown above Col are defective in the generation of this mRNA and contain a premature nonsense stop codon (blue frame). Ecotypes Col and those beneath have intact *RPF1* reading frames. Sap-0 has an intact *RPF1* reading frame, but strongly reduced amounts of the 1,538 nt dicistronic *nad4L-atp4* transcript (Fig. 1B).

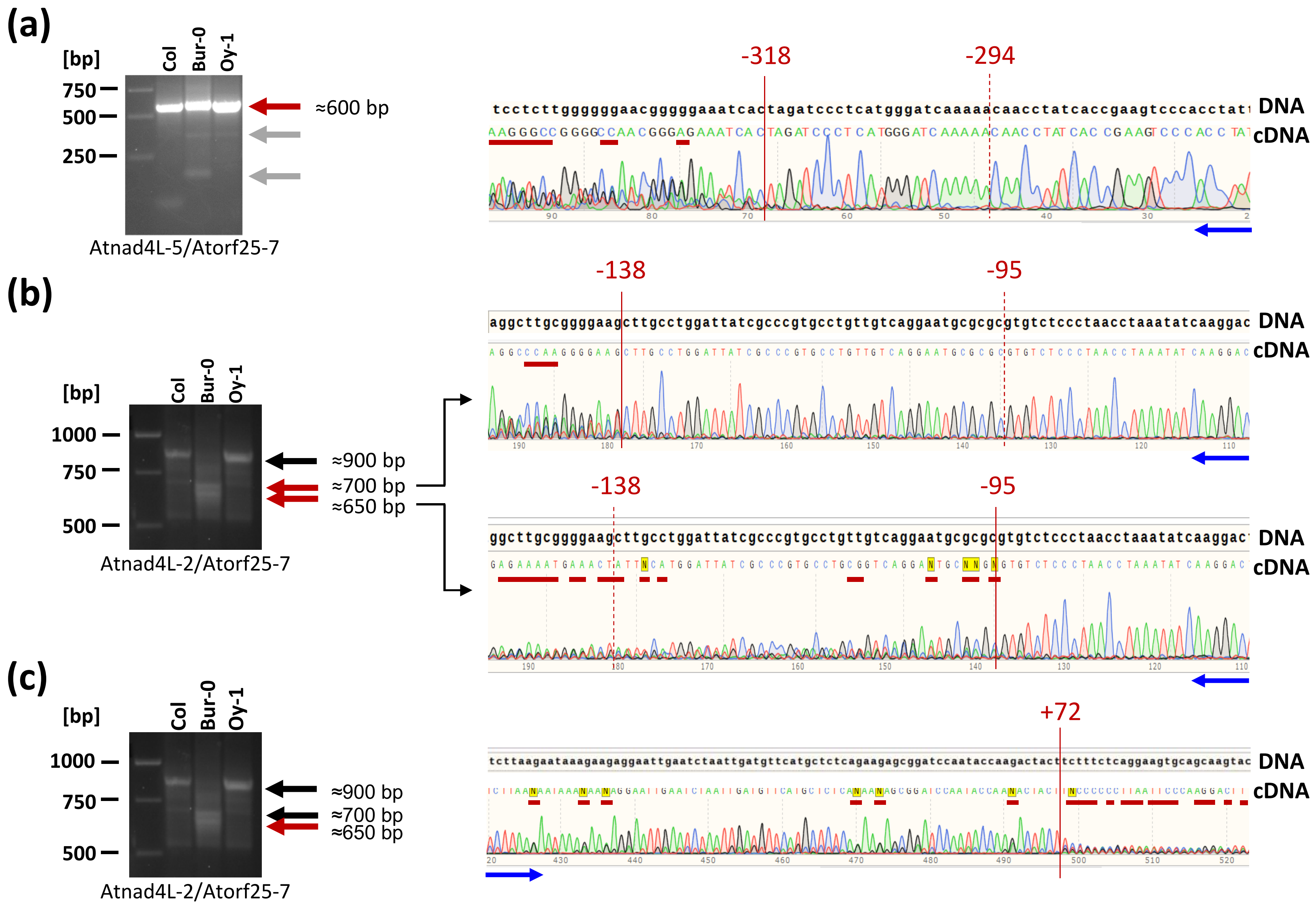

Supplemental Figure S3: CR-RT-PCR analysis of 5' and 3' ends of *nad4L-atp4* transcripts in Bur-0. Products amplified are shown in the images, approximate sizes given on the right hand side and oligonucleotides used are given below the images. Sequenced products are marked by red arrows. cDNA sequences (unambiguous nucleotide identities are marked by yellow boxes) are compared with the Arabidopsis mtDNA sequence (DNA, NC_037304), sequence divergences are indicated by red bars. Termini identified by a switch of the cDNA sequence quality indicated by underpeaks, unambiguous nucleotide identities or sizes of the peaks are marked by red vertical lines. Dashed red vertical lines mark ends identified in other sequencing reactions. Sequence analyses were performed with oligonucleotides Atnad4L-5 (**a**) Atnad4L-2 (**b**, both panels), and Atorf25-7 (**c**). Blue arrows indicate sequence direction.

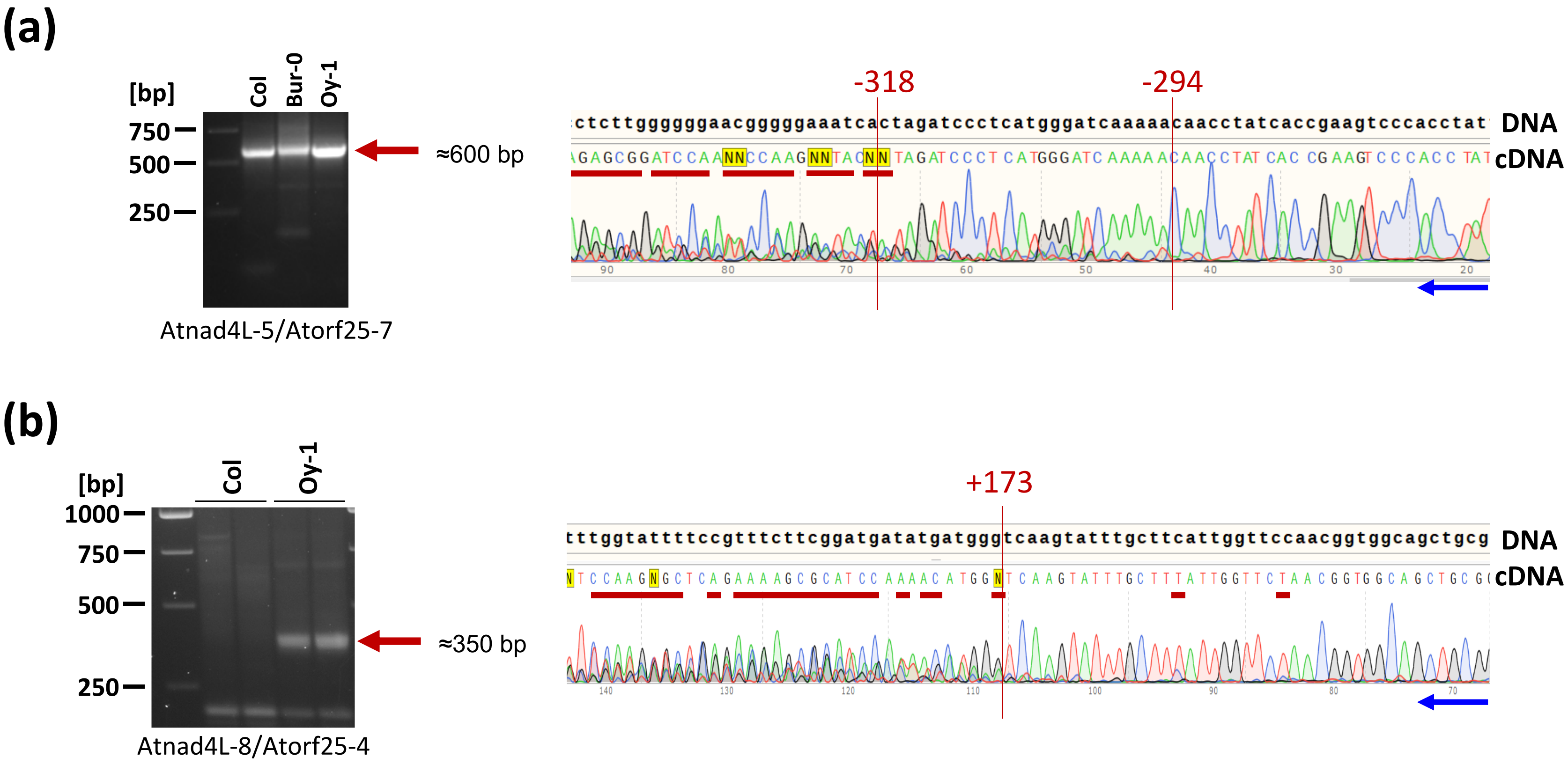

Supplemental Figure S4: CR-RT-PCR analysis of 5’ and 3’ ends of *nad4L-atp4* transcripts in Oy-1. Products amplified are shown in the images, approximate sizes given on the right hand side and oligonucleotides used are given below the images. Sequenced products are marked by red arrows. cDNA sequences (unambiguous nucleotide identities are marked by yellow boxes) are compared with the Arabidopsis mtDNA sequence (DNA, NC_037304), sequence divergences are indicated by red bars. Termini identified by a switch of the cDNA sequence quality indicated by underpeaks, unambiguous nucleotide identities or sizes of the peaks are marked by red vertical lines. Positions are given as in Supplemental Figure S1. Sequence analyses were performed with oligonucleotides Atnad4L-5 (**a**) and Atnad4L-8 (**b**). Blue arrows indicate sequence direction.

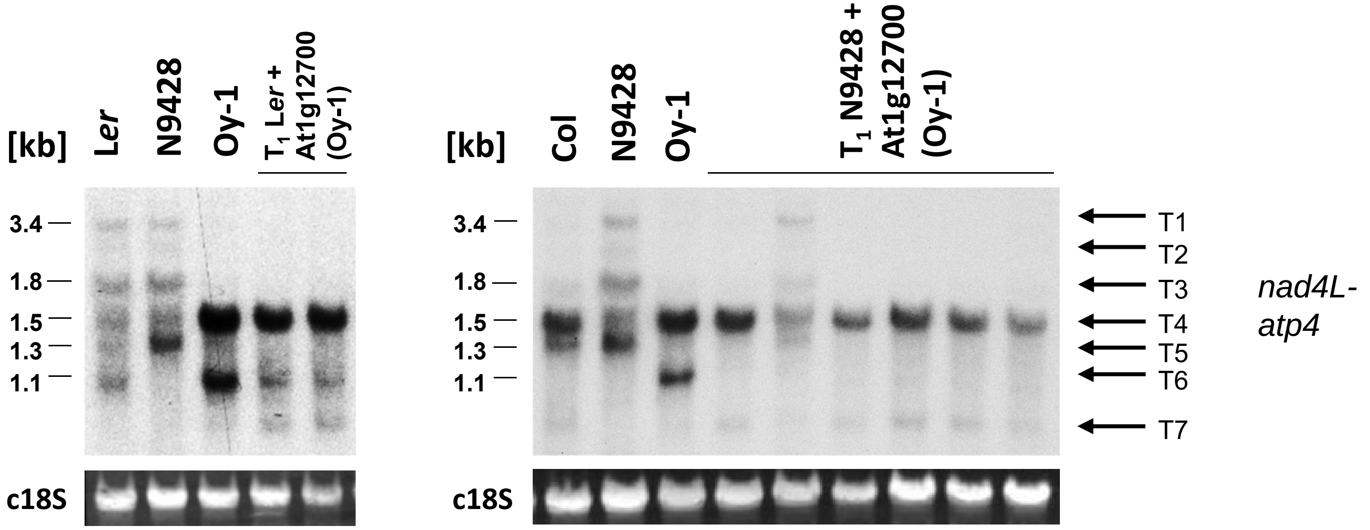

Supplemental Figure S5: In Oy-1, the generation of the 1,050 nt-long mRNA is independent of *RPF1***.** Northern blot hybridization of L*er* and N9428 plants containing the At1g12700 allele from Oy-1. Descriptions see legend to Fig. 1.

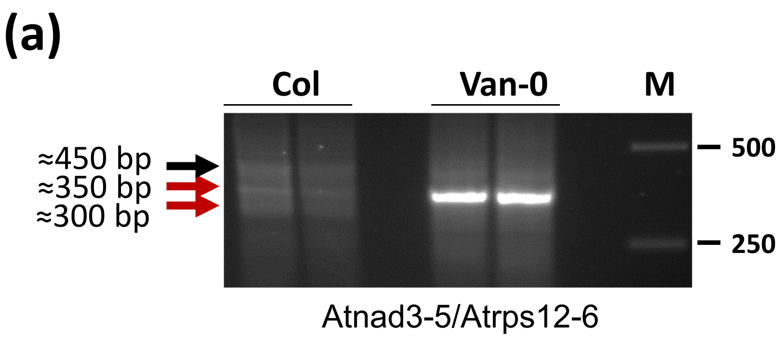

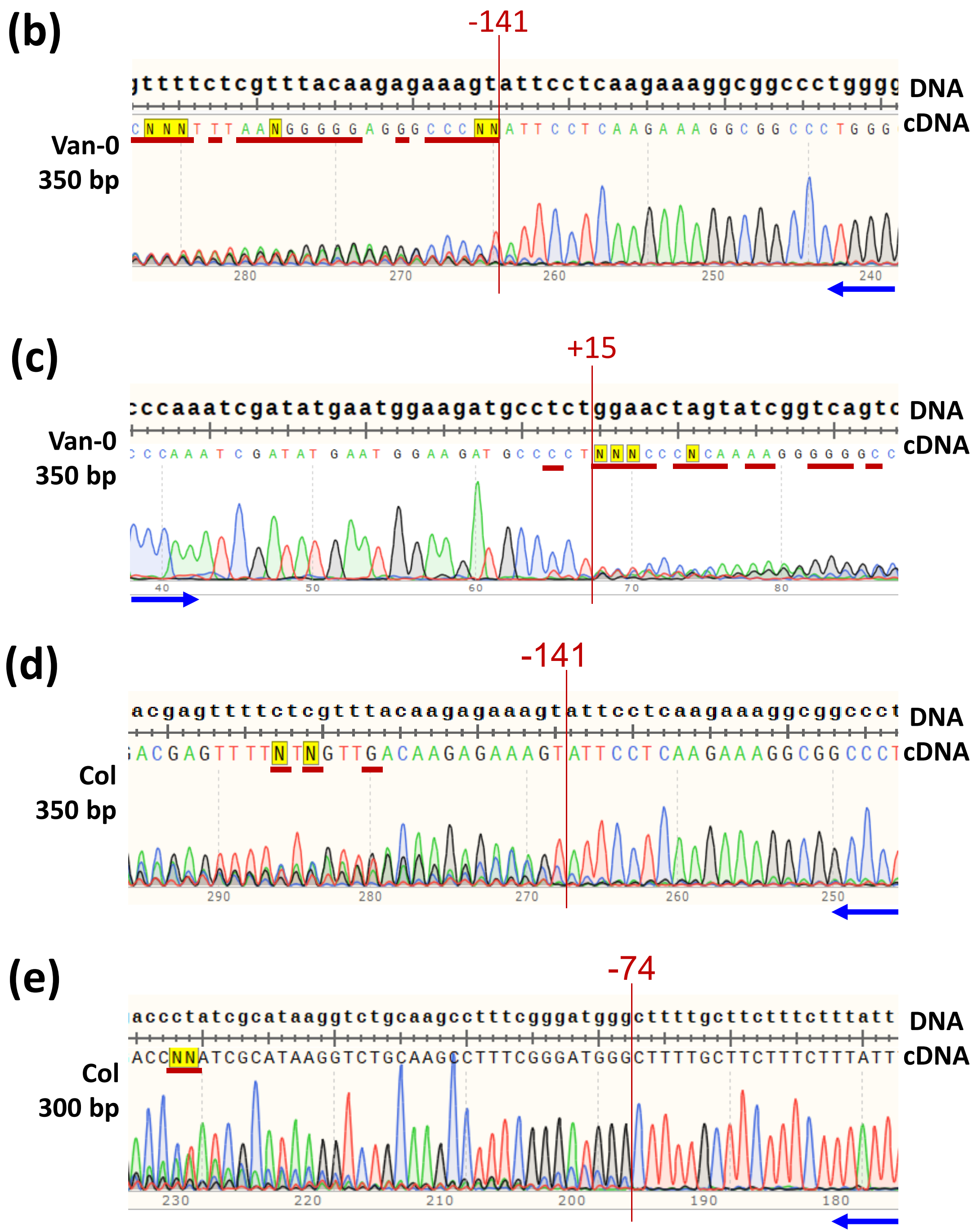

Supplemental Figure S6: CR-RT-PCR analysis of 5′ and 3′ ends of *nad3-rps12* transcripts in Van-0 and Col. Products amplified are highlighted in the left including approximate sizes. Primers used are given below the image. Sequenced products are marked by red arrows. cDNA sequences (unambiguous nucleotide identities are marked by yellow boxes) are compared with the Arabidopsis mtDNA sequence (DNA, NC_037304), sequence divergences are indicated by red bars. Termini identified by a switch of the cDNA sequence quality indicated by underpeaks, unambiguous nucleotide identities or sizes of the peaks are marked by red vertical lines. Positions are given as in Figure S1. Sequence analyses were performed with oligonucleotides Atnad3-5 (**b, d** and **e**) and Atrps12-6 (**c**). Blue arrows indicate sequence direction.

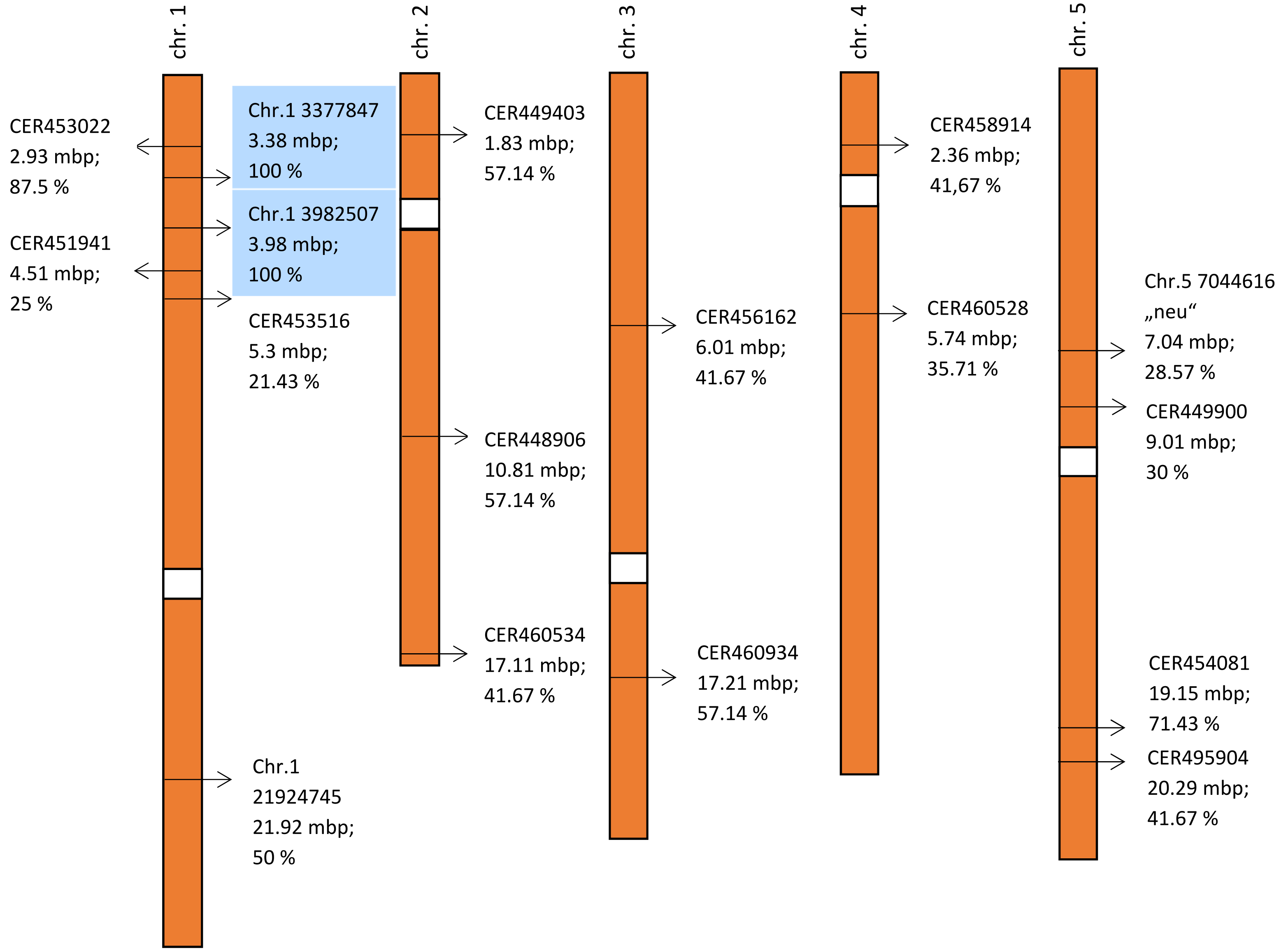

Supplemental Figure S7: Linkage analysis performed with a Col x Van-0 F_2 mapping population. Approximate positions of markers are given on the five chromosomes (orange boxes, white boxes indicate centromeres). Percentage [%] of Col alleles is indicated. Markers where exclusively Col alleles were found are highlighted by blue boxes.

```
Col         MRGLIQTRLLETGTLRTALFLSCYGRVFSSVSDGKGKVSYRERLRSGIVDIKEDDAVDLF   60
Blh-1       MRGLIQTRLLETGTLRTALFLSCYGRVFSSVSDGKGKVSYRERLRSGIVDIKKDDAVDLF   60
Got-7       MRGLIQTRLLETGTLRTALFLSCYGRVFSSVSDGKGKVSYRERLRSGIVDIKEDDAVDLF   60
Ra-0        MRGLIQTRLLQTGTLRTALFLSCYGRVFSSVSDGKGKVSYRERLRSGIVDIKEDDAVDLF   60
Tsu-1       MRGLIQTRLLQTGTLRTALFLSCYGRVFSSVSDGKGKVSYRERLRSGIVDIKEDDAVDLF   60
La-1        MRGLIQTRLLQTGTLRTALFLSCYGRVFSSVSDGKGKVSYRERLRSGIVDIKEDDAVDLF   60
Van-0       MRGLIQTRLLQTGTLRTALFLSCYGRVFSSVSDGKGKVSYRERLRSGIVDIKEDDAVDLF   60
            **********:*****************************************:*******

Col         QEMTRSRPRPRLIDFSRLFSVVARTKQYDLVLDLCKQMELKGIAHNLYTLSIMINCCCRC   120
Blh-1       QEMTRSRPRPSLIDFSRLFSVVARTKQYDLVLDLCKQMELKGIAHNLYTLSIMINCFCRC   120
Got-7       QEMTRSRPRPSLIDFSRLFSVVARTKQYDLVLDLCKQMELKGIAHNLYTLSIMINCFCRC   120
Ra-0        QEMTRSRPRPSLIDFSRLFSVVARTKQYDLVLDLCKQMELKGIAHNLYTLSIMINCFCRC   120
Tsu-1       QEMTRSRPRPSLIDFSRLFSVVARTKQYDLVLDLCKQMELKGIAHNLYTLSIMINCFCRC   120
La-1        QEMTRSRPRPSLIDFSRLFSVVARTKQYDLVLDLCKQMELKGIAHNLYTLSIMINCFCRC   120
Van-0       QEMTRSRPRPSLIDFSRLFSVVARTKQYDLVLDLCKQMELKGIAHNLYTLSIMINCFCRC   120
            ********** ********************************************* ***

Col         RKLSLAFSAMGKIIKLGYEPDTVTFSTLINGLCLEGRVSEALELVDRMVEMGHKPTLITL   180
Blh-1       RKLCLAFSAMGKIIKLGYEPDTVTFSTLINGLCLEGRVSEALELVDRMVEMGHKPTLITL   180
Got-7       RKLCLAFSAMGKIIKLGYEPDTVTFSTLINGLCLEGRVSEALELVDRMVEMGHKPTLITL   180
Ra-0        RKLCLAFSAMGKIIKLGYEPNTITFSTLINGLCLEGRVSEALELVDRMVEMGHKPTLITL   180
Tsu-1       RKLCLAFSAMGKIIKLGYEPNTITFSTLINGLCLEGRVSEALELVDRMVEMGHKPTLITL   180
La-1        RKLCLAFSAMGKIIKLGYEPNTITFSTLINGLCLEGRVSEALELVDRMVEMGHKPTLITL   180
Van-0       RKLCLAFSAMGKIIKLGYEPNTITFSTLINGLCLEGRVSEALELVDRMVEMGHKPTLITL   180
            ***.****************:*:************************************:

Col         NALVNGLCLNGKVSDAVLLIDRMVETGFQPNEVTYGPVLKVMCKSGQTALAMELLRKMEE   240
Blh-1       NTLVNGLCLNGKVSDAVLLIDRMVETGFQPNEVTYGPVLNVMCKSGQTALAMELLRKMEE   240
Got-7       NTLVNGLCLNGKVSDAVLLIDRMVETGFQPNEVTYGPVLNVMCKSAKLPLPWSCSERWKK   240
Ra-0        NTLVNGLCLNGKVSDAVLLIDRMVETGFQPNEVTYGPVLNVMCKSGQTALAMELLRKMEE   240
Tsu-1       NTLVNGLCLNGKVSDAVLLIDRMVETGFQPNEVTYGPVLNVMCKSGQTALAMELLRKMEE   240
La-1        NTLVNGLCLNGKVSDAVLLIDRMVETGFQPNEVTYGPVLNVMCKSGQTALAMELLRKMEE   240
Van-0       NTLVNGLCLNGKVSDAVLLIDRMVETGFQPNEVTYGPVLNVMCKSGQTALAMELLRKMEE   240
            *:*************************************:*****.:  *  .  .: ::

Col         R-KIKLDAVKYSIIIDGLCKDGSLDNAFNLFNEMEIKGFKADIIIYTTLIRGFCYAGRWD   299
Blh-1       R-KIKLDAVKYSIIIDGLCKHGSLDNAFNLFNEMEMKGFKADIIIYTTLIRGFCYAGRWD   299
Got-7       ERSSSMQSNTVSSL--MVCAKTGASTMH------------------------STFSMKWK   274
Ra-0        R-DIKLDAVKYSIIIDGLCIDGSLDDAFNLFNEMEIKGFKADIIIYTTLIRGFCYAGRWD   299
Tsu-1       R-DIKLDAVKYSIIIDGLCIDGSLDDAFNLFNEMEIKGFKADIIIYTTLIRGFCYAGRWD   299
La-1        R-DIKLDAVKYSIIIDGLCIDGSLDDAFNLFNEMEIKGFKADIIIYTTLIRGFCYAGRWD   299
Van-0       R-DIKLDAVKYSIIIDGLCIDGSLDDAFNLFNEMEIKGFKADIIIYTTLIRGFCYAGRWD   299
            . . .::: . * :   :* . . .  .                          :: :*.

Col         DGAKLLRDMIKRKITPDVVAFSALIDCFVKEGKLREAEELHKEMIQRGISPDTVTYTSLI   359
Blh-1       DGAKLLRDMIKRKITPDVVAFSALIDCFVKEGKLREAEELHKEMIQRGISPDTITYTSLI   359
Got-7       SKGSKLILL---STPLSLEAFVTLVDGMMVPSC*--------------------------   304
Ra-0        DGAKLLRDMIKRKITPDVVAFSALIDCFVKEGKLREAEELHKEMIQRGIAPDTITYTSLI   359
Tsu-1       DGAKLLRDMIKRKITPDVVAFSALIDCFVKEGKLREAEELHKEMIQRGIAPDTITYTSLI   359
La-1        DGAKLLRDMIKRKITPDVVAFSALIDCFVKEGKLREAEELHKEMIQRGIAPDTITYTSLI   359
Van-0       DGAKLLRDMIKRKITPDVVAFSALIDCFVKEGKLREAEELHKEMIQRGIAPDTITYTSLI   359
            . .. *  :   .   .: ** :*:* ::  .

Col         DGFCKENQLDKANHMLDLMVSKGCGPNIRTFNILINGYCKANLIDDGLELFRKMSLRGVV   419
Blh-1       DGFCKENQLDKANHMLDLMVSKGCGPNIRTFNILINGYCKANLIDDGLELFRKMSLRGVV   419
Got-7       ------------------------------------------------------------
Ra-0        DGFCKENHLDKANQMVDLMVSKGCDPNIRTFNILINGYCKANRIDDGLELFRKMSLRGVV   419
Tsu-1       DGFCKENHLDKANQMVDLMVSKGCDPNIRTFNILINGYCKANRIDDGLELFRKMSLRGVV   419
La-1        DGFCKENHLDKANQMVDLMVSKGCDPNIRTFNILINGYCKANRIDDGLELFRKMSLRGVV   419
Van-0       DGFCKENHLDKANQMVDLMVSKGCDPNIRTFNILINGYCKANRIDDGLELFRKMSLRGVV   419
            ************************************************************
```

Supplemental Fig. S8

```
Col         ADTVTYNTLIQGFCELGKLEVAKELFQEMVSRRVRPDIVSYKILLDGLCDNGEPEKALEI    479
Blh-1       ADTVTYNTLIQGFCELGKLEVAKELFQEMVSRRVRPDIVSYKILLDGLCDNGEPEKALEI    479
Got-7       ------------------------------------------------------------
Ra-0        ADTVTYNTLIQGFCELGKLNVAKELFQEMVSRKVPPNIVTYKILLDGLCDNGESEKALEI    479
Tsu-1       ADTVTYNTLIQGFCELGKLNVAKELFQEMVSRKVPPNIVTYKILLDGLCDNGESEKALEI    479
La-1        ADTVTYNTLIQGFCELGKLNVAKELFQEMVSRKVPPNIVTYKILLDGLCDNGESEKALEI    479
Van-0       ADTVTYNTLIQGFCELGKLNVAKELFQEMVSRKVPPNIVTYKILLDGLCDNGESEKALEI    479
            *******************:****************:**:************* ******

Col         FEKIEKSKMELDIGIYNIIIHGMCNASKVDDAWDLFCSLPLKGVKPDVKTYNIMIGGLCK    539
Blh-1       FEKIEKSKMELDIGIYNIIIHGMCNASKVDDAWDLFCSLPLKGVKPDVKTYNIMIGGLCK    539
Got-7       ------------------------------------------------------------
Ra-0        FEKIEKSKMELDIGIYNIIIHGMCNASKVDDAWDLFCSLPLKGVNPDVKTYNIMIGGLCK    539
Tsu-1       FEKIEKSKMELDIGIYNIIIHGMCNASKVDDAWDLFCSLPLKGVNPDVKTYNIMIGGLCK    539
La-1        FEKIEKSKMELDIGIYNIIIHGMCNASKVDDAWDLFCSLPLKGVNPDVKTYNIMIGGLCK    539
Van-0       FEKIEKSKMELDIGIYNIIIHGMCNASKVDDAWDLFCSLPLKGVNPDVKTYNIMIGGLCK    539
            ************************************************************

Col         KGSLSEADLLFRKMEEDGHSPNGCTYNILIRAHLGEGDATKSAKLIEEIKRCGFSVDAST    599
Blh-1       KGSLSEADLLFRKMEEDGHSPNGCTYNILIRAHLGESDATKSAKLIEEIKRCGFSVDAST    599
Got-7       ------------------------------------------------------------
Ra-0        KGSLSEADLLFRKMEEDGHSPNGCTYNILIRAHLGEGDATKSAKLIEEIKRCGFSVDAST    599
Tsu-1       KGSLSEADLLFRKMEEDGHSPNGCTYNILIRAHLGEGDATKSAKLIEEIKRCGFSVDAST    599
La-1        KGSLSEADLLFRKMEEDGHSPNGCTYNILIRAHLGEGDATKSAKLIEEIKRCGFSVDAST    599
Van-0       KGSLSEADLLFRKMEEDGHSPNGCTYNILIRAHLGEGDATKSAKLIEEIKRCGFSVDAST    599
            ************************************ ***********************

Col         VKMVVDMLSDGRLKKSFLDMLS*    621
Blh-1       VKMVVDMLSDGRLKKSFLDMLS*    621
Got-7       -----------------------
Ra-0        VKMVVDMLSDGRLKKSFLDMLS*    621
Tsu-1       VKMVVDMLSDGRLKKSFLDMLS*    621
La-1        VKMVVDMLSDGRLKKSFLDMLS*    621
Van-0       VKMVVDMLSDGRLKKSFLDMLS*    621
```

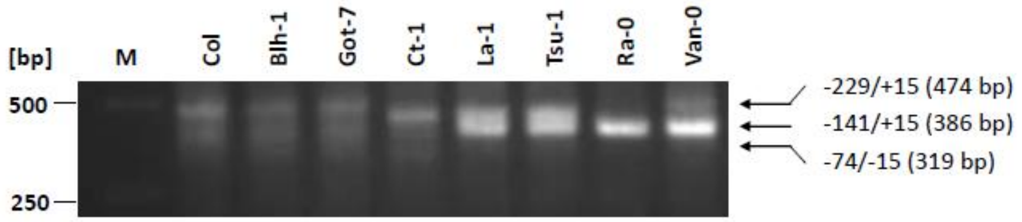

Supplemental Fig. S8: Amino acid sequences of *RPF8* alleles from different ecotypes with a Col-like (blue) and Van-0-like (blue) *nad3-rps12* mRNA phenotype. Vertical brown lines define pentatricopeptide repeats. Amino acid positions 2, 5 and 35 important for the function of PPR proteins are marked by yellow background. Bottom part: nad3-rps12 CR-RT-PCR product pattern (Ct-1 no amino acid sequence shown).

(a)

```
Col wt              MRGLIQTRLLETGTLRTALFLSCYGRVFSSVSDGKGKVSYRERLRSGIVDIKEDDAVDLF    60
C_Sp_RPF8_F-H       MRGLIQTRLLETGTLRTALFLSCYGRVFSSVSDGKGKVSYRERLRSGIVDIKEDDAVDLF    60
C_Sp_RPF8_KHJ1.23   MRGLIQTRLLETGTLRTALFLSCYGRVFSSVSDGKGKSPIERD*----------------    43
C_Sp_RPF8_KHJ1.24   MRGLIQTRLLETGTLRTALFLSCYGRVFSSVSDERESLL*--------------------    39

Col wt              QEMTRSRPRPRLIDFSRLFSVVARTKQYDLVLDLCKQMELKGIAHNLYTLSIMINCCCRC    120
C_Sp_RPF8_F-H       QEMTRSRPRPRLIDFSRLFSVVARTKQYDLVLDLCKQMELKGIAHNLYTLSIMINCCCRC    120

Col wt              RKLSLAFSAMGKIIKLGYEPDTVTFSTLINGLCLEGRVSEALELVDRMVEMGHKPTLITL    180
C_Sp_RPF8_F-H       RKLSLAFSAMGKIIKLGYEPDTVTFSTLINGLCLEGRVSEALELVDRMVEMGHKPTLITL    180

Col wt              NALVNGLCLNGKVSDAVLLIDRMVETGFQPNEVTYGPVLKVMCKSGQTALAMELLRKMEE    240
C_Sp_RPF8_F-H       NALVNGLCLNGKVSDAVLLIDRMVETGFQPNEVTYGPVLKVMCKSGQTALAMELLRKMEE    240

Col wt              RKIKLDAVKYSIIIDGLCKDGSLDNAFNLFNEMEIKGFKADIIIYTTLIRGFCYAGRWDD    300
C_Sp_RPF8_F-H       RKIKLDAVKYSIIIDGLCKDGSLDNAFNLFNEMEIKGFKADIIIYTTLIRGFCYAGRWDD    300

Col wt              GAKLLRDMIKRKITPDVVAFSALIDCFVKEGKLREAEELHKEMIQRGISPDTVTYTSLID    360
C_Sp_RPF8_F-H       GAKLLRDMIKRKITPGRGRFQRVNRLFCERGKASRG*-----------------------    336
```

(b)

```
Van-0 wt            MRGLIQTRLLQTGTLRTALFLSCYGRVFSSVSDGKGKVSYRERLRSGIVDIKEDDAVDLF    60
V_Sp_RPF8_KHJ22.1   MRGLIQTRLLQTGTLRTALFLSCYGRVFSSVSDGKRESLL*-------------------    40
V_Sp_RPF8_KHJ22.3   MRGLIQTRLLQTGTLRTALFLSCYGRVFSSVSDERE-SLL*-------------------    39
```

Supplemental Fig. S9: Amino acid sequences of CRISPR-Cas9-induced *RFP8* knockout alleles in Col (**a**) and Van-0 (**b**). Wild-type sequences are given only for those parts important for comparison. Vertical brown lines define pentatricopeptide repeats. Amino acid positions 2, 5 and 35 important for the function of PPR proteins are marked by yellow background. Amino acid identities deviating from the wild-type sequence due to CRISPR-Cas9-induced frame shifts are given in red.
